# Assessment of safety profile of secukinumab in real-world scenario using United States food and drug administration adverse event reporting system database

**DOI:** 10.1038/s41598-023-50013-7

**Published:** 2024-01-12

**Authors:** Vishnu Eshwar, Ashwin Kamath

**Affiliations:** Department of Pharmacology, Kasturba Medical College, Mangalore, Manipal Academy of Higher Education, Manipal, India

**Keywords:** Psoriasis, Adverse effects, Biological therapy

## Abstract

Secukinumab is an anti-IL-17 monoclonal antibody approved for treating psoriasis and various arthritides. A comprehensive evaluation of its safety, especially in a real-world setting, is necessary. This study aimed to describe the adverse events (AE) associated with secukinumab use using the United States Food and Drug Administration Adverse Event Reporting System (FAERS) database. FAERS data files containing AE reports from 2015 to 2021 were downloaded for data mining. Primary or secondary suspect medications indicated for psoriasis were identified and analyzed. Medical dictionary for regulatory activities (MedDRA version 24.1) was used to analyze the AE terms. To detect potential safety signals of AE from secukinumab use, disproportionality analysis was used. A total of 365,590 adverse event reports were identified; of these, 44,761 reports involved the use of secukinumab. Safety signals were identified for ocular infections and gastrointestinal adverse events at the standardised MedDRA query level. Safety signals for oral candidiasis, oral herpes, conjunctivitis, eye infections, and ulcerative colitis were identified at the preferred term level. The findings of our study are consistent with those of earlier studies, such as the increased risk of infections and inflammatory bowel disease. However, our study also identified additional safety signals that need to be further evaluated.

## Introduction

Biologics are genetically engineered drugs used as vaccines, therapeutic proteins, monoclonal antibodies, immunomodulators, and growth factors^1^. They can be composed of sugars, proteins, nucleic acids, or complex combinations of these substances^2^. These agents are generally well tolerated; however, serious and unexpected adverse drug reactions have been reported, affecting various organ systems at different stages of biologic therapy^3^. Such adverse drug events are often target-related and may be explained by the actions of the biological drug^4^.

Secukinumab is a fully humanized immunoglobulin G1 (IgG1) κ monoclonal antibody that inhibits the proinflammatory cytokine interleukin-17A (will be henceforth referred to as IL-17). The normal physiological and immunological functions of IL-17 include mucocutaneous defense^5^ and immunity against extracellular pathogens^6^. However, autoimmunity is observed with unregulated levels of IL-17, which are observed in conditions such as psoriasis and the spondylarthritis family of diseases^7,8^. Unregulated levels of IL-17 have also been documented in immunopathological conditions and cancer progression^9^. IL-17 as a novel pharmacological target has expanded the scope of drug development for various autoimmune diseases^10^, of which, secukinumab has proven efficacy in psoriasis, psoriatic arthritis, ankylosing spondylitis, non-radiographic axial spondylarthritis, and enthesis-related arthritis. Off-label use of secukinumab has also been reported in certain non-psoriatic dermatological conditions, such as hidradenitis suppurativa, pityriasis rubra pilaris, refractory spontaneous chronic urticaria, papulopustular rosacea, ABCA12 deficiency-related ichthyosis, and Bechet’s disease^11^. Secukinumab has elicited a favorable safety profile in clinical trials, but concerns have been raised with higher incidences of inflammatory bowel disease (IBD) and candida infection^12^. As the potential of IL-17 in other diseases is still being discovered, it is necessary to establish the safety profile of secukinumab comprehensively and address the adverse events that have occurred across all organ systems.

Post-marketing studies using real-world data are a powerful tool for evaluating the safety profile of medications. Spontaneous reporting databases can be used to detect safety signals, especially for serious and rare adverse events^13^. Performing a disproportionality analysis using the data from these databases can aid in establishing hypotheses of causality between the adverse event and the medication^14^. The United States Food and Drug Administration Adverse Event Reporting System (FAERS) is one such spontaneous reporting system that contains publicly accessible data and is a good source for such disproportionality studies. Our study aimed to identify the potential signals of adverse events of secukinumab in a real-world scenario using the FAERS database. To differentiate the adverse events which are common to all antipsoriatic biologics from those specific to secukinumab, we compared the adverse event data for secukinumab with those of other biological and non-biological drugs used for treating psoriasis.

## Methods

**Figure 1:**
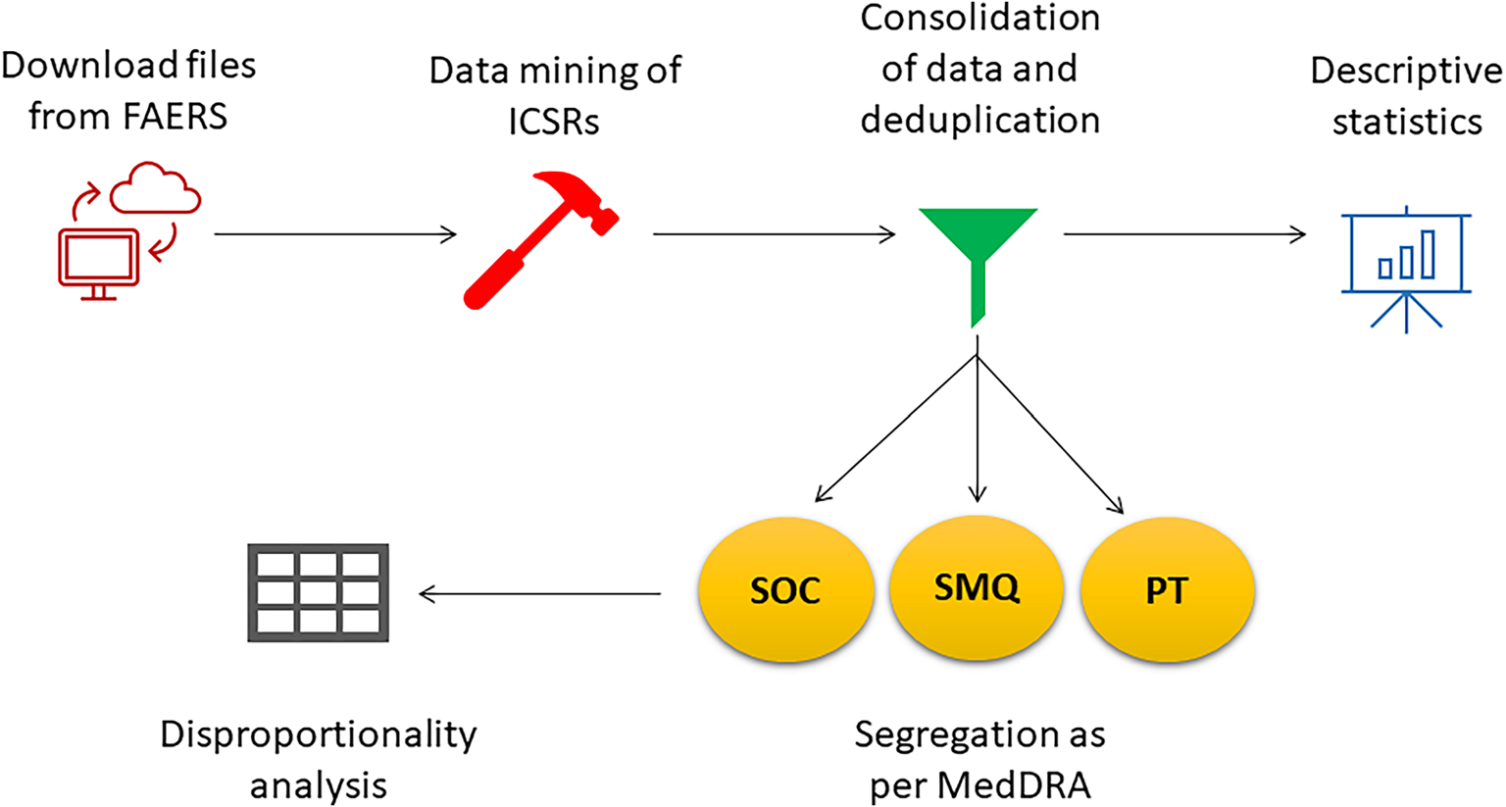
Study workflow for conducting disproportionality analysis. *FAERS* United States Food and Drug Administration adverse event reporting system, *ICSRs* individual case safety reports, *MedDRA* medical dictionary for regulatory activities, *SOC* system organ class, *SMQ* standardised MedDRA query, *PT* preferred term.

This is a retrospective, case-non-case study using the US FAERS database. FAERS is a public database containing adverse event reports submitted by healthcare professionals, drug manufacturers, and consumers. The database contains individual case safety reports (ICSR) in ASCII and XML file formats, and these are updated quarterly^15^. Each quarterly ASCII file contains dollar sign delimited text files containing information on demographics (DEMO), drugs (DRUG), indication for drug use (INDI), outcome of the adverse event (OUTC), the adverse event (REAC), report source (RPSR), and therapy dates (THER)^15^. Since secukinumab was released in the year 2015^16^, the ASCII quarterly files from the years 2015 to 2021 were downloaded from the FAERS website for data mining and analysis. The downloaded text files were then exported to Microsoft Excel® (2019). The methodology of our study is summarized in Fig. 1. The study protocol was approved by Kasturba Medical College Institutional Ethics Committee (IEC KMC MLR 10/2021/318).

### Indications and suspect medications

For this study, we included all ICSRs in which the suspected medications were indicated for treating psoriasis. The indication terms included were psoriasis, guttate psoriasis, pustular psoriasis, nail psoriasis, and erythrodermic psoriasis. The ICSRs containing these indication terms were identified from the INDI file. The Case IDs so obtained were used to identify the relevant ICSRs in the DRUG file. The drugs considered in this study were those recommended by the Joint American Academy of Dermatology-National Psoriasis Foundation Guidelines for the management of psoriasis; these consisted of biologics and systemic non-biologics that were approved by the US FDA for use in psoriasis^17,18^. The medications include biologics (adalimumab, brodalumab, certolizumab, etanercept, guselkumab, infliximab, ixekizumab, risankizumab, secukinumab, tildrakizumab and ustekinumab) and non-biologics (acitretin, apremilast, cyclosporine, methotrexate/methotrexate sodium). The ICSRs containing one or more of these drugs listed as the primary or secondary suspect medication were identified, and the information for these case reports was consolidated from the various files to obtain complete information on each ICSR.

### Identification and grouping of adverse event terms of interest

In the FAERS database, adverse events are listed using the Medical Dictionary for Regulatory Activities (MedDRA) Preferred Term (PT). PT is a distinct descriptor for a symptom, sign, disease diagnosis, therapeutic indication, investigation, surgical or medical procedure, and medical, social, or family history characteristic. There are 25,077 preferred terms coded by MedDRA. For analysis, the PTs were grouped based on the system organ class (SOC) and standardised MedDRA query (SMQ) terms. We used the MedDRA Desktop Browser (MedDRA Version 24.1) for grouping the PTs. Hence, each ICSR had one or more SOC and SMQ term(s) associated with it. SOCs function at the highest level of the adverse event term reporting hierarchy and provide the broadest concept for data retrieval based on aetiology, manifestation site, and purposes. An adverse event term may be assigned to multiple SOCs; only the primary SOC was considered for analysis. There are 27 SOC terms available, and disproportionality analysis was performed for each of these terms.

SMQs are re-grouping of adverse event terms, ordinarily, at the PT level, that relate to a defined medical condition or area of interest. There are 109 individual SMQ terms available in MedDRA version 24.1. Sometimes, a certain adverse event may fit the criteria of more than one SMQ. This is handled by MedDRA by assigning different levels (level 1–level 5) of SMQ to an adverse event. In addition, each SMQ term has a narrow or broad scope. A narrow scope is highly likely to represent the condition of interest, whereas a broad scope is less specific. To avoid ambiguity between different levels of SMQs and their scope, we considered SMQ Level 1 terms with narrow scope for analysis (Supplementary Table S1)^11^. We also performed analysis at the PT level. We included only those PTs in the analysis that were reported in ≥ 100 ICSRs with secukinumab as a suspect medication.

### Deduplication

In the data files, each ICSR is assigned a Primary ID, which is a concatenation of the case ID and the case version. To cleanse the consolidated data from duplicates, only the latest version of each ICSR was retained^15^. Furthermore, to avoid including duplicates of case reports that were reported by different sources and at different periods, thereby having been assigned different case IDs, deduplication was performed based on matching data for the following variables: event date, age, sex, reporter country, suspect drugs, and adverse event terms reported^19^. Following deduplication, the ICSRs were divided into three groups for conducting disproportionality analysis based on the suspected medication: the first group consisted of ICSRs wherein secukinumab was a suspect medication, irrespective of the presence of other anti-psoriatic drugs; the second group consisted of ICSRs containing rest of the biologics of interest; the third group consisted of ICSRs for non-biological agents as a suspect medication. The deduplication and group-wise consolidation of the ICSRs were done using RStudio (Version 1.4.1717).

### Disproportionality analysis

A disproportionality analysis determines whether there is disproportional reporting of a drug-adverse event combination compared with the occurrence of the adverse event with other drugs in the database^20^. Our study objective was to determine whether the reporting of any SOC term/SMQ term/PT was disproportionately high in association with secukinumab use. This was determined by calculating the proportional reporting ratio (PRR) and reporting odds ratio (ROR). PRR is the proportion of specific reactions (or groups of reactions) for drugs of interest, where the comparator is all other drugs in the database^21^. ROR is the ratio of an adverse event being reported in those who received the suspected medication to those who did not receive the suspected medication^22^. The calculation of disproportionality was performed using a two-by-two contingency table (Table 1). PRR and ROR are analogous to relative risk and odds ratio^22^. PRR > 2 and ROR > 2 with a lower bound 95% CI > 1 were considered significant^21^.

**Table 1:** Contingency table for disproportionality analysis.

	Adverse event of interest	All other adverse events	Total
Medication of interest	A	B	A + B
All other medications	C	D	C + D

Proportional reporting ratio = (A/A + B)/(C/C + D), Reporting odds ratio = (A/B)/(C/D).

### Statistical analysis

The clinical and demographic characteristics of the cases have been reported as median (interquartile range [IQR]). Categorical variables are reported as proportions and percentages. The data were analyzed using Microsoft Excel® (2019). In FAERS, age can be reported in multiple formats (year, decade, month, days); we only included those ICSRs where age was reported in years. The proportion of the male and female population along with their age characteristics was also analyzed. ICSRs with missing values for the variable of interest were excluded. Serious adverse event outcomes were acquired from the OUTC file; the outcomes could be death, life-threatening, hospitalization (initial or prolonged), disability, congenital anomaly, required intervention to prevent permanent impairment or damage (devices), and other serious (important medical events). A single ICSR may contain two or more reported outcomes; in such cases, each outcome was calculated separately.

## Results

### Demographic and clinical characteristics

**Figure 2:**
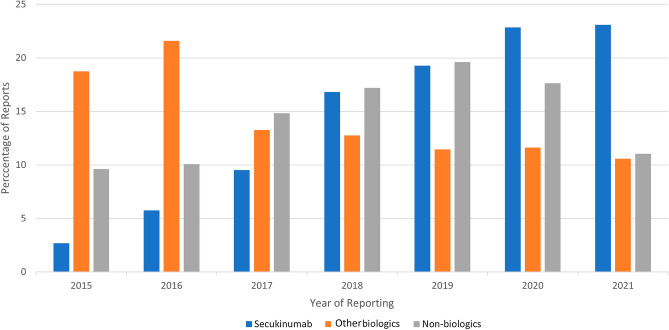
Yearly reporting of individual case safety reports (ICSR) of secukinumab, other biologics, and non-biologics, expressed as percentage of total in each group.

During the period 2015–2021, 365,590 adverse event reports were received by FAERS from the use of drugs indicated in systemic therapy for psoriasis. Following case ID deduplication and multifield deduplication, the total number of reports was 319,345 and 256,337, respectively. Among these, 44,761 (17.45%), 144,725 (56.42%), and 67,005 (26.12%) ICSRs were associated with secukinumab, other biologics, and non-biologics, respectively. The distribution of ICSRs throughout the years is depicted in Fig. 2.

The age and gender distribution of the 44,761 cases of adverse events associated with secukinumab use are shown in Table 2. The median age of the patients was 54 years (IQR, 44–63). Females represented 25,826 (56.49%) of the cases. 5124 (11.44%) cases were reported in the elderly population (≥ 65 years). The median duration of onset of AE from the time of initiation of secukinumab was 92 days (IQR, 21–326). Concomitant suspect medications were present in 4229 (9.4%) cases in the secukinumab group. Death was reported in 949 (2.1%) cases, 6591 (14.72%) were hospitalized, disability occurred in 586 (1.30%) cases, congenital anomaly in 14 (0.03%) cases, 11 (0.02%) cases required intervention, and 14,962 (33.42%) other serious adverse events were reported with the use of secukinumab. Of the 44,761 secukinumab-associated event reports, 8.24% were direct reports, 39.41% were expedited, and 52.35% were non-expedited; 58.75% reports were from consumers, 15.18% were from physicians, 2.79% were from pharmacists, and 23.28% were from others. Of the 144,725 aadverse event reports associated with other biologics, 9.30% were direct reports, 40.09% were expedited, and 50.61% were non-expedited; 49.64% reports were from consumers, 27.23% from physicians, 3.49% from pharmacists, and 19.64% from others. Of the 67,005 non-biologics-associated adverse event reports, 5.59% were direct reports, 17.06% were expedited, and 77.35% were non-expedited; 23.15% reports were from consumers, 18.96% from physicians, 16.74% from pharmacists, and 41.15% from others.

**Table 2:** Demographic characteristics of patients with psoriasis or related disorders with adverse events following use of a biologic or non-biologic drug.

Characteristics	Suspect drug		
	Secukinumab, N = 44,761	Other biologics, N = 144,725	Non-biologics, N = 67,005
Age in years, median (IQR)			
All	54 (44–63)	55 (44–63)	56 (46–64)
Female	54 (43–63)	54 (43–63)	56 (47–64)
Male	55 (43–63)	55 (45–64)	56 (47–64)
Unknown*	53 (41–61)	52 (38–63)	56 (40–65)
Gender, N (%)			
Female	25,826 (56.49)	82,690 (57.13)	42,452 (63.32)
Male	17,168 (38.35)	58,776 (40.16)	23,528 (35.11)
Unknown*	1767 (3.94)	3259 (2.71)	1025 (1.52)

*IQR* interquartile range.

*Information regarding gender not available in the adverse event report.

**Figure 3:**
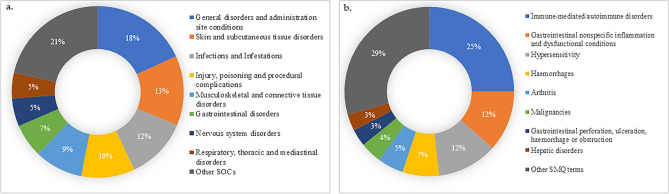
Most common adverse events reported in patients with psoriasis or related disorders following secukinumab use categorized according to the (**a**) MedDRA system organ class (**b**) standardised MedDRA query. *MedDRA* medical dictionary for regulatory activities.

**Figure 4:**
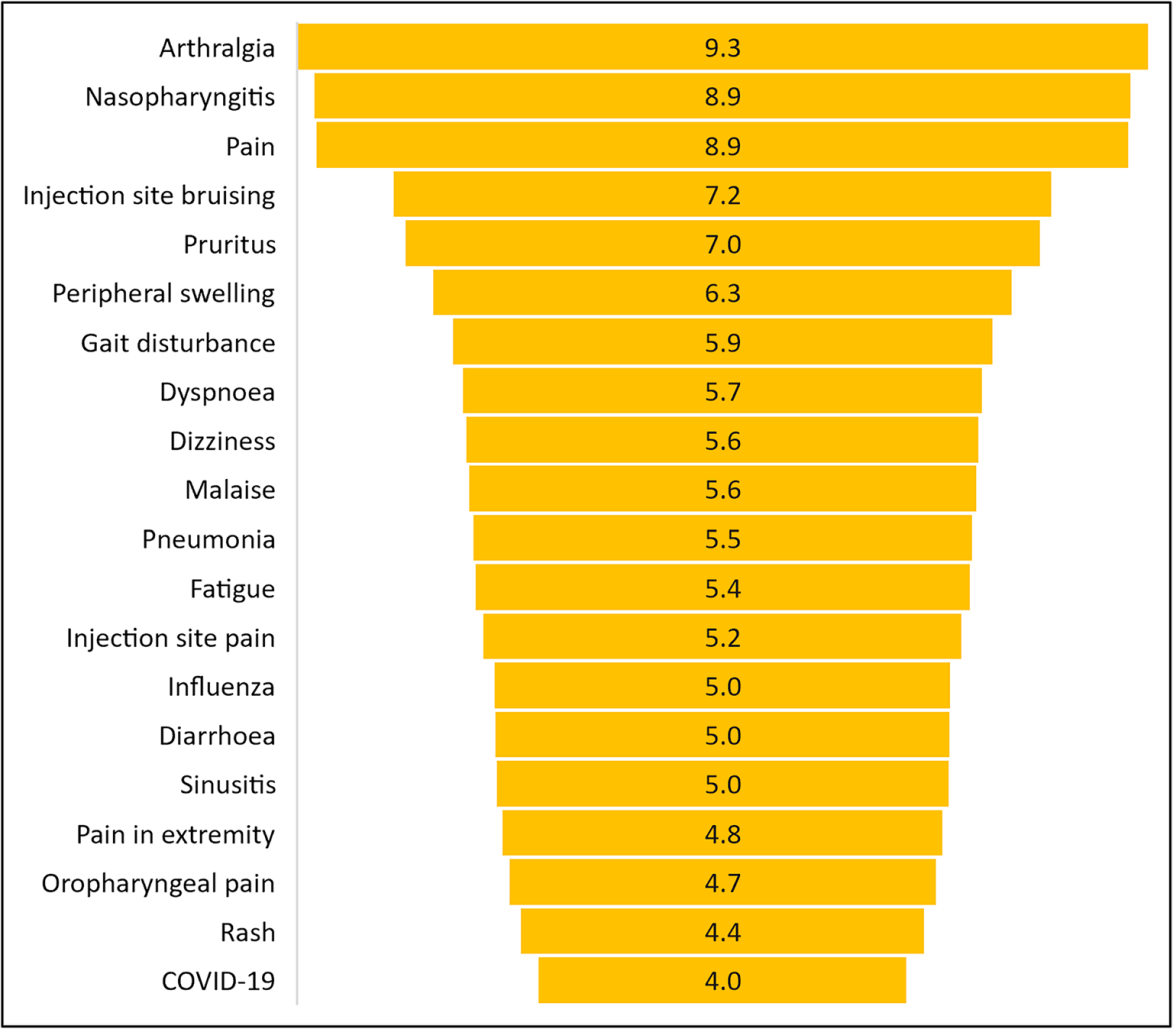
Most common MedDRA preferred terms reported in patients with psoriasis or related disorders following secukinumab use expressed as percentage of total number of ICSRs (N = 44,761; excludes the preferred terms psoriasis, psoriatic arthropathy, and drug ineffective which were reported in 36.50%, 16.72%, and 14.76% of ICSRs, respectively). *MedDRA* medical dictionary for regulatory activities, *ICSR* individual case safety report.

The most commonly reported adverse events at the SOC level (Fig. 3a) were general disorders and administrative site conditions (18%) and skin and subcutaneous tissue disorder (13%); at the SMQ level (Fig. 3b), immune-mediated/autoimmune disorders (25%) and gastrointestinal nonspecific inflammation and dysfunctional conditions (12%). The most commonly reported adverse events at the preferred term level are shown in Fig. 4.

### Disproportionality analysis

#### System organ class terms

**Figure 5:**
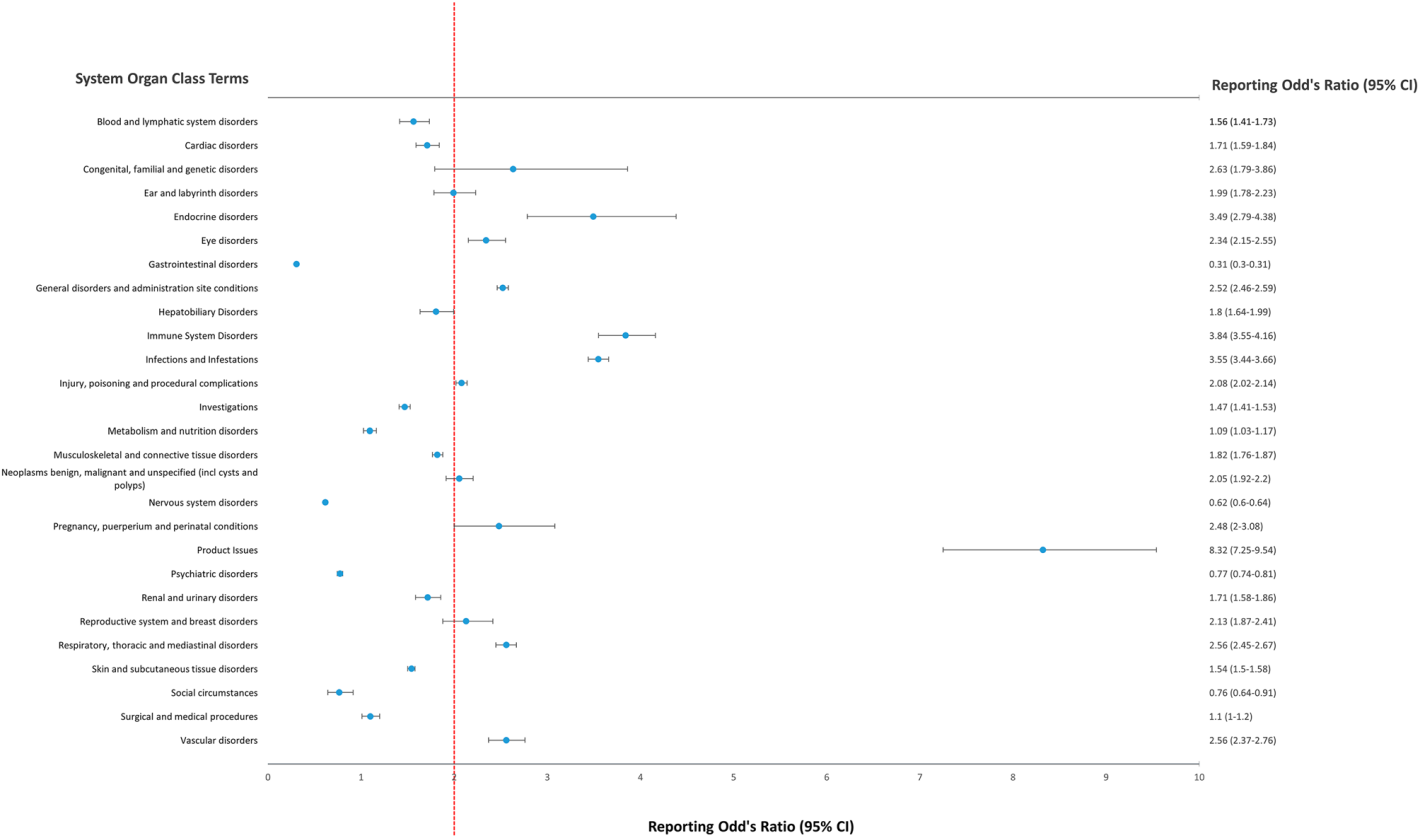
Reporting odds ratio of adverse events at the MedDRA system organ class level reported in patients with psoriasis or related disorders receiving secukinumab or non-biologics. *MedDRA* medical dictionary for regulatory activities, *CI* confidence interval.

No disproportionate signal was observed when comparing secukinumab with other biologics (Supplementary Table S2). When compared with non-biologics (Fig. 5, Supplementary Table S3), disproportionate reporting (ROR) was observed in immune system disorders 3.84 (95% CI, 3.55–4.16); infections and infestations 3.55 (3.44–3.66); endocrine disorders 3.49 (2.79–4.38); congenital, familial and genetic disorders 2.63 (1.79–3.86); vascular disorders 2.56 (2.37–2.76); respiratory, thoracic and mediastinal disorders 2.56 (2.45–2.67); general disorders and administration site conditions 2.52 (2.46–2.59); pregnancy, puerperium and perinatal conditions 2.48 (2–3.08); and eye disorders 2.34 (2.15–2.55).

#### Standardised MedDRA query terms

**Figure 6:**
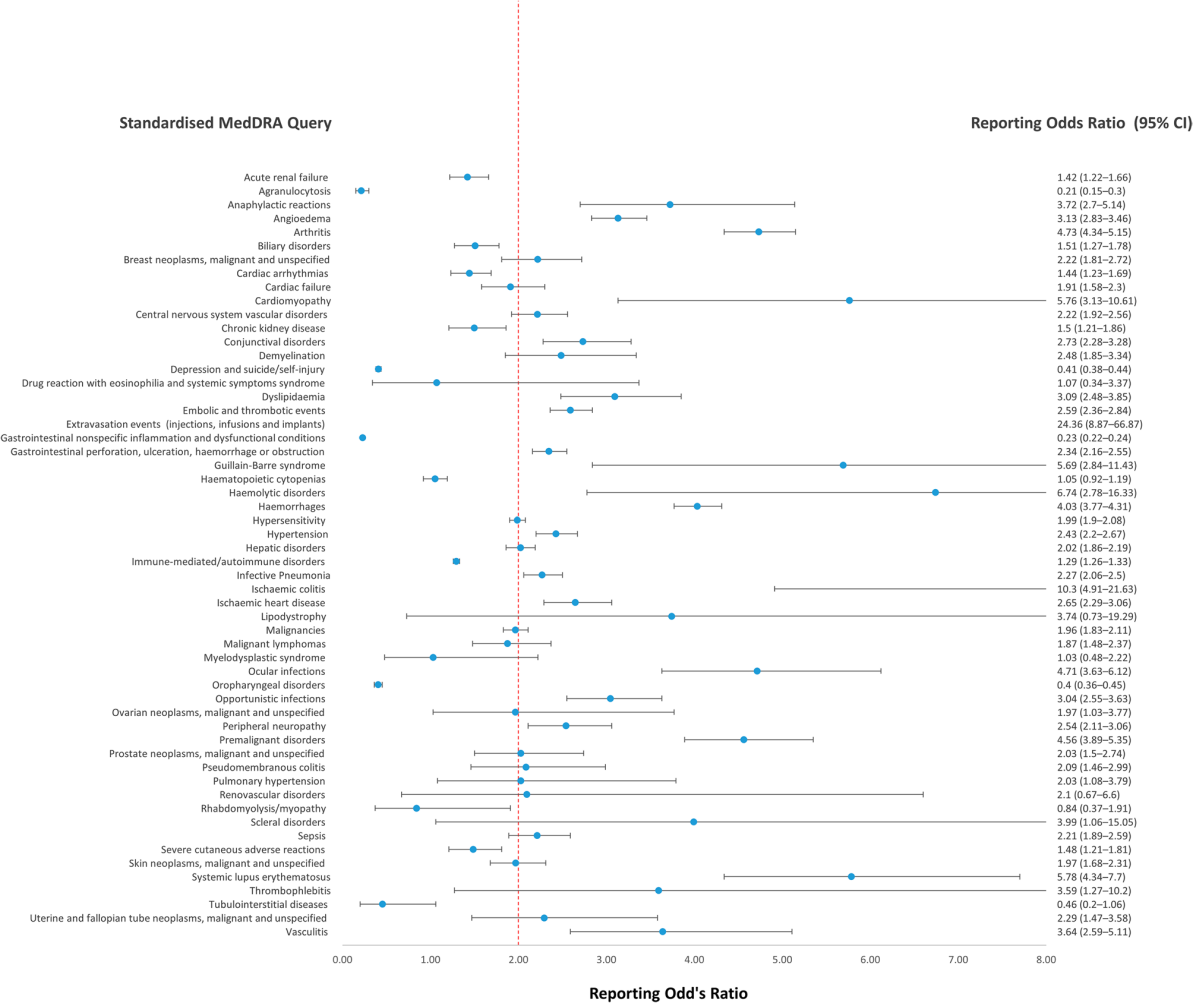
Reporting odds ratio of adverse events at the standardised MedDRA query level reported in patients with psoriasis or related disorders receiving secukinumab or non-biologics. *MedDRA* medical dictionary for regulatory activities, *CI* confidence interval.

A disproportionality signal (ROR) was observed in ocular infections 2.31 (95% CI, 1.96–2.74) and gastrointestinal nonspecific inflammation and dysfunctional conditions 2.16 (2.08–2.24) compared with the other biologics group (Supplementary Table S4). When compared with non-biologics (Fig. 6, Supplementary Table S5), several SMQs showed disproportionality. The SMQ terms with ROR > 3 are extravasation events (injections, infusions and implants) 24.36 (8.87–66.87), ischaemic colitis 10.3 (4.91–21.63), haemolytic disorders 6.74 (2.78–16.33), systemic lupus erythematosus 5.78 (4.34–7.7), cardiomyopathy 5.76 (3.13–10.61), Guillain–Barre syndrome 5.69 (2.84–11.43), arthritis 4.73 (4.34–5.15), ocular infections 4.71 (3.63–6.12), premalignant disorders 4.56 (3.89–5.35), and haemorrhages 4.03 (3.77–4.31).

#### Preferred terms

The results of disproportionality analysis for preferred terms comparing secukinumab with other biologics and non-biologics are given in Supplementary Table S6. Adverse events having the strongest signals (ROR > 7) when compared with other biologics included concomitant disease aggravated with ROR 210.57 (95% CI, 78.43–565.32), macule 15.99 (12.49–20.47), pigmentation disorder 13.79 (9.59–19.82), movement disorder 13.54 (10.03–18.28), blood pressure systolic increased 11.73 (7.78–17.7), anosmia 10.56 (8.11–13.75), rebound psoriasis 9.91 (7.29–13.48), product prescribing error 9.41 (7.92–11.18), hypokinesia 8.47 (6.27–11.43), and aphthous ulcer 7.32 (5.18–10.37).

The strongest disproportionality signals when compared with other non-biologics included (ROR > 50) injection site bruising 216.91 (95% CI, 145.14–324.16), injection site haemorrhage 173.61 (98.34–306.52), injection site pruritus 210.02 (67.46–653.9), injection site pain77.22 (58.01–102.8), device malfunction 88.3 (43.89–177.62), product storage error 84.96 (37.9–190.48), therapeutic response shortened 54.41 (30.65–96.56), injection site erythema 47.8 (28.58–79.93), injection site swelling 51.28 (27.34–96.18), and device issue 51.07 (26.32–99.11).

## Discussion

Our study assessed the safety profile of secukinumab using the FAERS database from 2015 to 2021. A steady increase in adverse event reports was observed year-on-year, which probably reflects the increasing use of the drug since its approval. Since an array of biological and non-biological drugs are used in the treatment of psoriasis, ICSRs containing secukinumab were compared with those of other biologicals approved for psoriasis and non-biological agents. Age distribution was similar among all the groups. Females represented approximately 55% of all safety reports. We performed disproportionality analysis at three levels: SOC, SMQ, and PT. Previous studies either focused on a specific adverse effect of secukinumab or analyzed the ICSRs irrespective of the indication for which the drug was used^23^.

Our study, which specifically looked at ICSRs reported in patients with psoriasis or related disorders, showed that, based on SOC, no disproportional reporting was observed when comparing secukinumab with other biological drugs. However, disproportional reporting was seen in comparison to non-biologics in the following: infection and infestation, immune system disorder, eye disorders, vascular disorder, endocrine disorder, general and administrative site disorders, and respiratory, thoracic, and mediastinal disorders. On analysis based on SMQ, although a large number of disproportionate reporting was seen compared to non-biologics, the disproportionate reporting was confined to gastrointestinal nonspecific inflammation and dysfunction conditions compared with other biologicals. These findings suggest that most of the adverse events with disproportionate reporting compared to the non-biologics are common to all biologics because there are no significant differences in the events reported with secukinumab and other biologics, except for certain gastrointestinal conditions and ocular disorders.

Comparison at the PT level showed disproportionate reporting of several adverse event terms. In comparison with other biologicals, AE terms suggestive of infection, such as pyrexia, COVID-19, oral candidiasis, and erysipelas; AE terms suggestive of gastrointestinal disorders, such as diarrhoea, abdominal discomfort, haematochezia, diarrhoea haemorrhagic, colitis, colitis ulcerative, inflammatory bowel disease, irritable bowel syndrome; AE terms for ocular disorders, such as conjunctivitis and ocular hyperaemia; and AE terms suggestive of musculoskeletal/neurological disorders, such as musculoskeletal stiffness, spinal pain, dysstasia, ankylosing spondylitis, movement disorder, and hypokinesia, showed disproportionately high reporting. Other terms with high reporting included blood pressure systolic increased, panniculitis, angioedema, and liver injury.

Among the ocular disorders, uveitis is of particular interest. Clinical trials and post-marketing studies have shown an exposure-adjusted incident rate of 0.01–0.02 per 100 patient-years in patients with psoriasis^24,25^. Our study revealed disproportionate reporting of uveitis (ROR 13.98 [8.81–22.17]) in comparison with non-biologic drugs. It is unclear whether these cases can be ascribed to drug-induced infection, inflammation, or abnormal immune reaction. A disproportionate reporting of ocular infections was seen in comparison with other biologics as well as non-biologics; there is scarce literature on the occurrence of ocular infections associated with secukinumab or other interleukin-17 inhibitors. However, there are a few case reports of ocular infection caused by a virus (*Herpes Simplex*)^26^*,* fungus (*Histoplasma capsulatum*) ^27^, and bacteria (*Staphylococcus aureus* and nontuberculous mycobacterium)^28,29^ manifesting as keratitis, scleritis, endophthalmitis, and uveitis, respectively. Interestingly, all the causative organisms from these case reports are known to be opportunistic pathogens, which further suggests the immunosuppressive actions of secukinumab to have possibly played a role in worsening or manifesting these infections. This fact is also supported by the finding in our study of decreased immune response with an ROR of 7.91 (6.69–9.35).

The risk of infections with IL-17 inhibitors and other monoclonal antibodies is well documented. IL-17 is a proinflammatory cytokine involved in extracellular immunity and mucocutaneous defense^6^. Our study showed disproportionate reporting of coronavirus infection in comparison with both other biologics and non-biologics. However, existing literature suggests secukinumab to be safe, and the incidence was similar to that in the general population^30,31^. Two case reports describe patients having recovered from COVID-19 without having to discontinue treatment^32,33^. In addition, a study showed that lower angiotensin converting enzyme-2 levels due to IL-17 inhibition may lower the risk of contracting COVID-19^34^. These studies suggest that secukinumab is safe even during the active infection phase of COVID-19. However, more studies are needed to identify the true associations between monoclonal antibodies and their use in patients with COVID-19. Higher reporting of fungal infections, especially candidiasis (oral candidiasis and oropharyngeal candidiasis), was found in our study. The incidence of candidiasis with IL-17 inhibitors is well established in the literature^35^. The imbalance of IL-17 secretion is associated with higher incidences of candidiasis and *Staphylococcus aureus* infection^6^. Disproportionate signals were also identified with other oral adverse events, including aphthous ulcer, oral pain, oropharyngeal pain, oropharyngeal discomfort, glossodynia, tonsillitis, mouth ulcerations, dysphagia, stomatitis, swollen tongue, swollen lip, and dry throat. Whether these events were associated infections is unknown. Post-marketing studies in patients with psoriasis have estimated the incidence of candidiasis to be 2.2/100 patient-years^36^. Opportunistic infections are another concern with the use of monoclonal antibodies for the treatment of autoimmune diseases^37^. A Vigibase study found a strong relationship between secukinumab use and herpes simplex virus with an ROR of 4.80 (3.78–6.10)^38^. Our study also found a disproportionate signal for oral herpes (ROR, 2.67 [2.33–3.06]). No safety signal was established for tuberculosis in any of the study groups. Besides the findings regarding coronavirus and herpes virus, the reporting of other viral infections was not significantly high, except for influenza in comparison to the non-biologics.

No new risks were identified with respect to malignancies apart from a signal for basal cell carcinoma in comparison with the non-biologics group. No increased risk was seen in pooled clinical trials and post-marketing studies of patients on secukinumab, with an exposure-adjusted incident rate of 0.83/100 patient-years in the psoriasis group^39^. However, it must be noted that psoriasis by itself is a risk factor for malignancy, particularly keratinocyte cancer and lymphomas^40^. The role of IL-17 is controversial and attributed to both tumour immunity and tumour proliferation^41^.

The SMQ vasculitis was another safety signal detected with the use of secukinumab. On the PT level, hypersensitivity vasculitis did not show increased reporting. Case reports exist in the literature on the occurrence of vasculitis with secukinumab: one case of IgA-vasculitis^42^ and another of cutaneous vasculitis with gut involvement^43^. A disproportionate signal was also found for Bechet’s disease. Case reports of occurrence of Bechet’s disease are available^44,45^, and interestingly enough, secukinumab has been used off-label for Bechet’s disease^46^. A clinical trial of secukinumab on aortic vascular inflammation in patients with psoriasis showed a neutral effect on aortic vascular inflammation and biomarkers of cardiometabolic disease^47^. The occurrence of Bechet’s syndrome may be a paradoxical effect of IL-17 inhibitors, and more studies are required to understand the actions of IL-17 inhibition on the blood vessels. A disproportionate signal was identified in comparison with the combined drug group for angina pectoris and decreased systolic blood pressure. The incidence of MACE in clinical settings has been low, with exposure-adjusted incidence rates of 0.3–0.4/100 patient-years in psoriasis^25,36^. IL-17 is seen to be involved in most cardiac and metabolic chronic diseases, including obesity and non‐alcoholic fatty liver^48^. However, the cardiometabolic effects of IL-17 inhibition are not well established.

The use of biologics may affect normal immune function and response, precipitating autoimmune conditions^49^. Our study showed increased reporting of rebound psoriasis, ankylosing spondylitis, and rheumatoid nodules; however, it is unclear whether these represent true events or are just associations, given that secukinumab is indicated in these conditions. Angioedema at the SMQ and PT levels was observed to have a safety signal. The incidence of angioedema is not well established in the literature. There exists a case report of a patient having recurrent angioedema with severe urticaria^50^. Another study also concluded that IL-17 inhibitors have a higher likelihood of precipitating immunological adverse events^51^.

Safety signals were identified for several dermatological events at the PT level, such as macule, pigmentation disorder, skin lesion, skin fissures, skin exfoliation, skin plaque, erysipelas, scratch, skin haemorrhage, pain of skin, and blister. At the SOC level (skin and subcutaneous tissue disorders), no disproportionality signal was observed in comparison with other biologics and non-biologics. At the SMQ level, the reporting of extravasation events is particularly high; however, no disproportionate reporting of severe cutaneous adverse reactions is seen in comparison to other biologics and non-biologics. Dermatological manifestations such as pemphigus, eczema, psoriasiform eruptions, hidradenitis suppurativa, and atopic dermatitis are observed with the use of secukinumab^11^. The role of IL-17 in the pathogenesis of the aforementioned adverse events has been described^52^; however, these seemingly paradoxical inflammation of the skin may have developed due to the immune imbalance between Th1 and Th2 secretions, implying that the manifestations may be due to Th2-derived secretions^53^. Further investigations are necessary to delineate whether the higher rates of dermatological adverse events observed are drug-induced or due to the disease for which the drug is indicated.

Pharyngitis, nasopharyngitis, and cough were common adverse events in clinical trials along with headache, pruritis, diarrhoea, arthralgia, back pain, and upper respiratory tract infections^54^. IL-17A and IL-17F have the potential to control the influx of neutrophils in conditions such as asthma, lung allograft rejection, and cystic fibrosis^55^. There is a need for studies to understand the inhibition of IL-17 in the respiratory system. The findings of our study also indicate a higher reporting of these common respiratory adverse events with secukinumab.

Gastrointestinal adverse events are a concern with the use of IL-17 inhibitors. In the current study, compared with biologics, secukinumab showed higher reporting of the SMQ gastrointestinal nonspecific inflammation and dysfunctional conditions; compared with non-biologics, disproportionate reporting was seen for gastrointestinal perforation, ulceration, haemorrhage, or obstruction, and ischaemic colitis. A phase-II clinical trial was stopped due to the worsening of Crohn’s disease^56^. In this trial, four of seven drug-related adverse events were worsening of Crohn’s disease. A retrospective study of pooled data from 21 clinical trials showed low incidences (< 1%) of inflammatory bowel disease across all indications^57^. Another meta-analysis also arrived at similar conclusions (2.4 cases per 1000 patient‐year)^58^, and the same was concluded from a real-world study^59^. Analysis at the PT level showed disproportionate reporting of colitis ulcerative, inflammatory bowel disease, and irritable bowel syndrome, among others, with secukinumab compared with biologics. These findings are in line with that of another study conducted using the WHO database, Vigibase, where inflammatory bowel disease and colitis had an ROR value of 3.36 (3.19–3.55)^60^. The cause of IBD in patients receiving IL-17 inhibitors may be due to the protective role of IL-17 in the gastric mucosa^61^. The magnitude and mechanism behind the manifestation of IBD need more clarity, and physicians must be well-informed of this association prior to prescribing anti-IL-17 agents. In addition, other gastrointestinal adverse events such as gastroenteritis, haematochezia, and haemorrhagic diarrhoea also showed safety signals. Disproportionate reporting was also observed for liver injury and pyelonephritis. The drug label mentions that phase 3 trials have shown elevation of hepatic transaminases, similar to the comparator etanercept group but more than that reported with placebo^16^. FDA medical review of secukinumab mentions grade 1 elevations in serum creatinine, more than placebo or etanercept group, which are transient and reversible, not requiring treatment discontinuation^62^. In comparison with placebo-treated patients, the changes in laboratory values from baseline or outliers were not considered to be clinically significant. Ageusia, anosmia, ascites, eating disorder, and speech disorders have also shown safety signals. The role of IL-17 in these cases, if at all a causal relationship exists, is yet to be determined.

Our study described the safety profile of secukinumab by mining real-world data from the FAERS database. For the analysis, we compared the adverse event reports with secukinumab use with those reported with other biologics and non-biologics used to treat psoriasis; this helped identify disproportionate signaling and place it in the context of other drugs. Accordingly, many secukinumab-associated events with disproportionate reporting were identified as those common to all antipsoriatic biologics, whereas a few were exclusive to secukinumab. We included primary and secondary suspect medications indicated in the treatment of psoriasis; hence, the study results describe the safety concerns with secukinumab therapy in psoriasis more accurately. In addition, our method of extensive deduplication reduces the likelihood of duplicates existing in our study data. Thus, our study differs from earlier studies evaluating secukinumab safety, which either focused on specific categories of adverse events^60,63,64^ and did not use specific comparators or additional deduplication steps^65^.

Our study has limitations. Our study was limited to analyzing the adverse events of secukinumab in patients with psoriasis with or without comorbidities. Hence, adverse events reported more frequently in patients with other indications may have been missed. The number of reports based on which a disproportionate reporting is identified can vary to a large extent, from fewer than hundred to thousands of reports, based on several factors. Because the number of reports containing the various preferred terms varied widely in our study, this can affect the study results. Also, a significant disproportionality statistic does not necessarily indicate causality given the presence of multiple factors that could have contributed to the occurrence of the adverse event. Given the limited data available in the ICSRs, it is not possible to adjust for these factors. There are known limitations with the use of FAERS data, considering that it is a spontaneous reporting AE database, which makes the findings hypothesis-generating but not confirmatory. Time on market is another potential confounder given that secukinumab, relative to other biologics and non-biologics used as comparators, is a new drug. All the non-biologics, except for apremilast, were approved for the treatment of psoriasis in the US prior to 2000. However, the findings of this study, considered together with earlier literature from clinical trials and real-world data, provide a more complete picture of the safety of secukinumab and areas requiring further studies. The presence of duplicate case reports is an important drawback of FAERS. We screened for duplicate reports by comparing multiple data fields. Although this approach can identify a large number of duplicate reports, it cannot identify all such reports, which require the implementation of complex algorithms for identification.

## Conclusion

Our study supports the safety findings of secukinumab described in earlier literature with regard to candidiasis, oral herpes, inflammatory bowel disease, and injection site reactions. In addition, new safety signals were identified, such as eye infection, synovitis, pyelonephritis, ascites, spinal pain, dysstasia, and hypokinesia. While many reported events seem to be common to all antipsoriatic biologics, in that no significant differences were observed between secukinumab and other biologicals, some were seen to have disproportionate reporting with secukinumab, such as ischemic colitis, ocular infections, gastrointestinal nonspecific inflammation and dysfunctional conditions, and gastrointestinal perforation, ulceration, haemorrhage or obstruction. Further clinical studies are required to determine the relatedness of these events to secukinumab and their characteristics.

### Supplementary Information

Supplementary Tables.

## Data Availability

All data pertaining to this study is based on the data available in the US FDA Adverse Event Reporting System database which is open to public.
