# Supplementary material for: Assessment of safety profile of secukinumab in real-world scenario using United States food and drug administration adverse event reporting system database
Source: Sci Rep. 2024 Jan 12;14:1222. doi: 10.1038/s41598-023-50013-7 (PMC10786882; doi:10.1038/s41598-023-50013-7)
Supplement: Supplementary file 1 — Supplementary Tables. [file 41598_2023_50013_MOESM1_ESM.docx]

**Supplementary Information**

**Assessment of Safety Profile of Secukinumab in Real-World Scenario Using United States Food and Drug Administration Adverse Event Reporting System Database**

Vishnu Eshwar,^1^ Ashwin Kamath^1^

^1^Department of Pharmacology, Kasturba Medical College, Mangalore, Manipal Academy of Higher Education, Manipal, India.

**Supplementary Table S1.** List of standardised MedDRA queries included for disproportionality analysis

|  | **Standardised MedDRA query terms** |  | **Standardised MedDRA query terms** |
| --- | --- | --- | --- |
| 1 | Acute renal failure | 29 | Immune-mediated/autoimmune disorders |
| 2 | Agranulocytosis | 30 | Infective pneumonia |
| 3 | Anaphylactic reaction | 31 | Ischaemic colitis |
| 4 | Angioedema | 32 | Ischaemic heart disease |
| 5 | Arthritis | 33 | Lipodystrophy |
| 6 | Biliary disorders | 34 | Malignancies |
| 7 | Breast neoplasms, malignant and unspecified | 35 | Malignant lymphomas |
| 8 | Cardiac arrhythmias | 36 | Myelodysplastic syndrome |
| 9 | Cardiac failure | 37 | Ocular infections |
| 10 | Cardiomyopathy | 38 | Opportunistic infections |
| 11 | Central nervous system vascular disorders | 39 | Oropharyngeal disorders |
| 12 | Chronic kidney disease | 40 | Ovarian neoplasms, malignant and unspecified |
| 13 | Conjunctival disorders | 41 | Peripheral neuropathy |
| 14 | Demyelination | 42 | Premalignant disorders |
| 15 | Depression and suicide/self-injury | 43 | Prostate neoplasms, malignant and unspecified |
| 16 | Drug reaction with eosinophilia and systemic symptoms syndrome | 44 | Pseudomembranous colitis |
| 17 | Dyslipidemia | 45 | Pulmonary hypertension |
| 18 | Embolic/Thromboembolic events. | 46 | Renovascular disorders |
| 19 | Extravasation events | 47 | Rhabdomyolysis/myopathy |
| 20 | Gastrointestinal nonspecific inflammation and dysfunctional conditions | 48 | Scleral disorders |
| 21 | Gastrointestinal perforation, ulceration, haemorrhage or obstruction | 49 | Sepsis |
| 22 | Guillain-Barre syndrome | 50 | Severe cutaneous adverse reactions |
| 23 | Haematopoietic cytopenias | 51 | Skin neoplasms, malignant and unspecified |
| 24 | Haemolytic disorders | 52 | Systemic lupus erythematosus |
| 25 | Haemorrhages | 53 | Thrombophlebitis |
| 26 | Hepatic disorders | 54 | Tubulointerstitial diseases |
| 27 | Hypersensitivity | 55 | Uterine and fallopian tube neoplasms, malignant and unspecified |
| 28 | Hypertension | 56 | Vasculitis |

**Supplementary Table S2.** Disproportionality analysis of adverse events at the system organ class level reported in patients with psoriasis or related disorders receiving secukinumab or other biologics

| **System Organ Class** | **With secukinumab** | | **With other biologics** | | **Disproportionality statistic** | |
| --- | --- | --- | --- | --- | --- | --- |
|  | **Number of AEs of interest** | **Number of other AEs** | **Number of AEs of interest** | **Number of other AEs** | **PRR** | **ROR (95% CI)** |
| Blood and lymphatic system disorders | 775 | 43986 | 2256 | 142468 | 1.11 | 1.11 (1.02–1.21) |
| Cardiac disorders | 1470 | 43291 | 5158 | 139566 | 0.92 | 0.92 (0.87–0.97) |
| Congenital, familial and genetic disorders | 72 | 44689 | 236 | 144488 | 0.99 | 0.99 (0.76–1.28) |
| Ear and labyrinth disorders | 693 | 44068 | 1805 | 142919 | 1.24 | 1.25 (1.14–1.36) |
| Endocrine disorders | 251 | 44510 | 588 | 144136 | 1.38 | 1.38 (1.19–1.6) |
| Eye disorders | 1345 | 43416 | 4434 | 140290 | 0.98 | 0.98 (0.92–1.04) |
| Gastrointestinal disorders | 7372 | 37389 | 15122 | 129602 | 1.58 | 1.69 (1.64–1.74) |
| General disorders and administration site conditions | 21081 | 23680 | 61253 | 83471 | 1.11 | 1.21 (1.19–1.24) |
| Hepatobiliary Disorders | 892 | 43869 | 3084 | 141640 | 0.94 | 0.93 (0.87–1.01) |
| Immune System Disorders | 2167 | 42594 | 4495 | 140229 | 1.56 | 1.59 (1.51–1.67) |
| Infections and Infestations | 13519 | 31242 | 32734 | 111990 | 1.34 | 1.48 (1.45–1.52) |
| Injury, poisoning and procedural complications | 11705 | 33056 | 35888 | 108836 | 1.05 | 1.07 (1.05–1.1) |
| Investigations | 4651 | 40110 | 12764 | 131960 | 1.18 | 1.2 (1.16–1.24) |
| Metabolism and nutrition disorders | 1666 | 43095 | 4340 | 140384 | 1.24 | 1.25 (1.18–1.32) |
| Musculoskeletal and connective tissue disorders | 10478 | 34283 | 27607 | 117117 | 1.23 | 1.3 (1.26–1.33) |
| Neoplasms benign, malignant and unspecified (including cysts and polyps) | 1903 | 42858 | 8872 | 135852 | 0.69 | 0.68 (0.65–0.72) |
| Nervous system disorders | 6100 | 38661 | 18378 | 126346 | 1.07 | 1.08 (1.05–1.12) |
| Pregnancy, puerperium and perinatal conditions | 218 | 44543 | 1065 | 143659 | 0.66 | 0.66 (0.57–0.76) |
| Product issues | 1321 | 43440 | 7342 | 137382 | 0.58 | 0.57 (0.54–0.6) |
| Psychiatric disorders | 3333 | 41428 | 7904 | 136820 | 1.36 | 1.39 (1.34–1.45) |
| Renal and urinary disorders | 1307 | 43454 | 3768 | 140956 | 1.12 | 1.13 (1.06–1.2) |
| Reproductive system and breast disorders | 584 | 44177 | 1953 | 142771 | 0.97 | 0.97 (0.88–1.06) |
| Respiratory, thoracic and mediastinal disorders | 5659 | 39102 | 12340 | 132384 | 1.48 | 1.55 (1.5–1.61) |
| Skin and subcutaneous tissue disorders | 15222 | 29539 | 31956 | 112768 | 1.54 | 1.82 (1.78–1.86) |
| Social circumstances | 187 | 44574 | 1770 | 142954 | 0.34 | 0.34 (0.29–0.39) |
| Surgical and medical procedures | 831 | 43930 | 8744 | 135980 | 0.31 | 0.29 (0.27–0.32) |
| Vascular disorders | 1838 | 42923 | 5389 | 139335 | 1.1 | 1.11 (1.05–1.17) |

AE, adverse event; ROR, reporting odds ratio; PRR, proportional reporting ratio; CI, confidence interval.

**Supplementary Table S3.** Disproportionality analysis of adverse events at the system organ class level reported in patients with psoriasis or related disorders receiving secukinumab or non-biologics

| **System Organ Class** | **With secukinumab** | | **With non-biologics** | | **Disproportionality statistic** | |
| --- | --- | --- | --- | --- | --- | --- |
|  | **Number of AEs of interest** | **Number of other AEs** | **Number of AEs of interest** | **Number of other AEs** | **PRR** | **ROR (95% CI)** |
| Blood and lymphatic system disorders | 775 | 43986 | 747 | 66258 | 1.55 | 1.56 (1.41–1.73) |
| Cardiac disorders | 1470 | 43291 | 1305 | 65700 | 1.69 | 1.71 (1.59–1.84) |
| Congenital, familial and genetic disorders | 72 | 44689 | 41 | 66964 | **2.63** | **2.63 (1.79–3.86)** |
| Ear and labyrinth disorders | 693 | 44068 | 525 | 66480 | 1.98 | 1.99 (1.78–2.23) |
| Endocrine disorders | 251 | 44510 | 108 | 66897 | **3.48** | **3.49 (2.79–4.38)** |
| Eye disorders | 1345 | 43416 | 875 | 66130 | **2.3** | **2.34 (2.15–2.55)** |
| Gastrointestinal disorders | 7372 | 37389 | 26285 | 40720 | 0.42 | 0.31 (0.3–0.31) |
| General disorders and administration site conditions | 21081 | 23680 | 17483 | 49522 | 1.81 | 2.52 (2.46–2.59) |
| Hepatobiliary Disorders | 892 | 43869 | 747 | 66258 | 1.79 | 1.8 (1.64–1.99) |
| Immune System Disorders | 2167 | 42594 | 876 | 66129 | **3.7** | **3.84 (3.55–4.16)** |
| Infections and Infestations | 13519 | 31242 | 7284 | 59721 | **2.78** | **3.55 (3.44–3.66)** |
| Injury, poisoning and procedural complications | 11705 | 33056 | 9757 | 57248 | 1.8 | 2.08 (2.02–2.14) |
| Investigations | 4651 | 40110 | 4907 | 62098 | 1.42 | 1.47 (1.41–1.53) |
| Metabolism and nutrition disorders | 1666 | 43095 | 2286 | 64719 | 1.09 | 1.09 (1.03–1.17) |
| Musculoskeletal and connective tissue disorders | 10478 | 34283 | 9649 | 57356 | 1.63 | 1.82 (1.76–1.87) |
| Neoplasms benign, malignant and unspecified (including cysts and polyps) | 1903 | 42858 | 1418 | 65587 | **2.01** | **2.05 (1.92–2.2)** |
| Nervous system disorders | 6100 | 38661 | 13667 | 53338 | 0.67 | 0.62 (0.6–0.64) |
| Pregnancy, puerperium and perinatal conditions | 218 | 44543 | 132 | 66873 | **2.47** | **2.48 (2–3.08)** |
| Product issues | 1321 | 43440 | 244 | 66761 | 8.1 | 8.32 (7.25–9.54) |
| Psychiatric disorders | 3333 | 41428 | 6310 | 60695 | 0.79 | 0.77 (0.74–0.81) |
| Renal and urinary disorders | 1307 | 43454 | 1155 | 65850 | 1.69 | 1.71 (1.58–1.86) |
| Reproductive system and breast disorders | 584 | 44177 | 414 | 66591 | **2.11** | **2.13 (1.87–2.41)** |
| Respiratory, thoracic and mediastinal disorders | 5659 | 39102 | 3588 | 63417 | **2.36** | **2.56 (2.45–2.67)** |
| Skin and subcutaneous tissue disorders | 15222 | 29539 | 16800 | 50205 | 1.36 | 1.54 (1.5–1.58) |
| Social circumstances | 187 | 44574 | 366 | 66639 | 0.76 | 0.76 (0.64–0.91) |
| Surgical and medical procedures | 831 | 43930 | 1133 | 65872 | 1.1 | 1.1 (1–1.2) |
| Vascular disorders | 1838 | 42923 | 1103 | 65902 | **2.49** | **2.56 (2.37–2.76)** |

AE, adverse event; ROR, reporting odds ratio; PRR, proportional reporting ratio; CI, confidence interval.

**Supplementary Table S4.** Disproportionality analysis of adverse events at the standardised MedDRA query level reported in patients with psoriasis or related disorders receiving secukinumab or other biologics

| **Standardised MedDRA Query** | **With secukinumab** | | **With other biologics** | | **Disproportionality statistic** | |
| --- | --- | --- | --- | --- | --- | --- |
|  | **Number of AEs of interest** | **Number of other AEs** | **Number of AEs of interest** | **Number of other AEs** | **PRR** | **ROR (95% CI)** |
| Acute renal failure | 321 | 44440 | 1021 | 143703 | 1.02 | 1.02 (0.9–1.15) |
| Agranulocytosis | 40 | 44721 | 119 | 144605 | 1.09 | 1.09 (0.76–1.56) |
| Anaphylactic reactions | 129 | 44632 | 278 | 144446 | 1.50 | 1.5 (1.22–1.85) |
| Angioedema | 1209 | 43552 | 1988 | 142736 | 1.97 | 1.99 (1.85–2.14) |
| Arthritis | 2164 | 42597 | 4383 | 140341 | 1.60 | 1.63 (1.54–1.71) |
| Biliary disorders | 275 | 44486 | 1286 | 143438 | 0.69 | 0.69 (0.61–0.79) |
| Breast neoplasms, malignant and unspecified | 226 | 44535 | 684 | 144040 | 1.07 | 1.07 (0.92–1.24) |
| Cardiac arrhythmias | 303 | 44458 | 1027 | 143697 | 0.95 | 0.95 (0.84–1.08) |
| Cardiac failure | 248 | 44513 | 880 | 143844 | 0.91 | 0.91 (0.79–1.05) |
| Cardiomyopathy | 50 | 44711 | 167 | 144557 | 0.97 | 0.97 (0.71–1.33) |
| Central nervous system vascular disorders | 471 | 44290 | 1633 | 143091 | 0.93 | 0.93 (0.84–1.03) |
| Chronic kidney disease | 166 | 44595 | 686 | 144038 | 0.78 | 0.78 (0.66–0.93) |
| Conjunctival disorders | 329 | 44432 | 547 | 144177 | 1.94 | 1.95 (1.7–2.24) |
| Demyelination | 116 | 44645 | 445 | 144279 | 0.84 | 0.84 (0.69–1.03) |
| Depression and suicide/self–injury | 761 | 44000 | 1798 | 142926 | 1.37 | 1.37 (1.26–1.5) |
| Drug reaction with eosinophilia and systemic symptoms syndrome | 5 | 44756 | 12 | 144712 | 1.35 | 1.35 (0.47–3.82) |
| Dyslipidaemia | 245 | 44516 | 608 | 144116 | 1.30 | 1.3 (1.12–1.51) |
| Embolic and thrombotic events | 1215 | 43546 | 4084 | 140640 | 0.96 | 0.96 (0.9–1.03) |
| Extravasation events (injections, infusions and implants) | 65 | 44696 | 308 | 144416 | 0.68 | 0.68 (0.52–0.89) |
| Gastrointestinal nonspecific inflammation and dysfunctional conditions | 5133 | 39628 | 8181 | 136543 | **2.03** | **2.16 (2.08–2.24)** |
| Gastrointestinal perforation, ulceration, haemorrhage or obstruction | 1437 | 43324 | 2464 | 142260 | 1.89 | 1.92 (1.79–2.05) |
| Guillain–Barre syndrome | 38 | 44723 | 93 | 144631 | 1.32 | 1.32 (0.91–1.93) |
| Haematopoietic cytopenias | 400 | 44361 | 863 | 143861 | 1.50 | 1.5 (1.33–1.69) |
| Haemolytic disorders | 27 | 44734 | 58 | 144666 | 1.51 | 1.51 (0.95–2.38) |
| Haemorrhages | 3073 | 41688 | 9024 | 135700 | 1.10 | 1.11 (1.06–1.16) |
| Hypersensitivity | 5038 | 39723 | 10531 | 134193 | 1.55 | 1.62 (1.56–1.67) |
| Hypertension | 1067 | 43694 | 1896 | 142828 | 1.82 | 1.84 (1.71–1.98) |
| Hepatic disorders | 1382 | 43379 | 4115 | 140609 | 1.09 | 1.09 (1.02–1.16) |
| Immune-mediated/autoimmune disorders | 10963 | 33798 | 20889 | 123835 | 1.70 | 1.92 (1.87–1.97) |
| Infective Pneumonia | 1051 | 43710 | 3656 | 141068 | 0.93 | 0.93 (0.87–0.99) |
| Ischaemic colitis | 55 | 44706 | 97 | 144627 | 1.83 | 1.83 (1.32–2.55) |
| Ischaemic heart disease | 509 | 44252 | 1707 | 143017 | 0.96 | 0.96 (0.87–1.06) |
| Lipodystrophy | 5 | 44756 | 20 | 144704 | 0.81 | 0.81 (0.3–2.15) |
| Malignancies | 1805 | 42956 | 7508 | 137216 | 0.78 | 0.77 (0.73–0.81) |
| Malignant lymphomas | 155 | 44606 | 562 | 144162 | 0.89 | 0.89 (0.75–1.07) |
| Myelodysplastic syndrome | 11 | 44750 | 38 | 144686 | 0.94 | 0.94 (0.48–1.83) |
| Ocular infections | 232 | 44529 | 325 | 144399 | **2.31** | **2.31 (1.96–2.74)** |
| Oropharyngeal disorders | 343 | 44418 | 5015 | 139709 | 0.22 | 0.22 (0.19–0.24) |
| Opportunistic infections | 374 | 44387 | 1101 | 143623 | 1.10 | 1.1 (0.98–1.24) |
| Ovarian neoplasms, malignant and unspecified | 21 | 44740 | 93 | 144631 | 0.73 | 0.73 (0.45–1.17) |
| Peripheral neuropathy | 299 | 44462 | 849 | 143875 | 1.14 | 1.14 (1–1.3) |
| Premalignant disorders | 615 | 44146 | 1524 | 143200 | 1.30 | 1.31 (1.19–1.44) |
| Prostate neoplasms, malignant and unspecified | 100 | 44661 | 454 | 144270 | 0.71 | 0.71 (0.57–0.88) |
| Pseudomembranous colitis | 71 | 44690 | 181 | 144543 | 1.27 | 1.27 (0.96–1.67) |
| Pulmonary hypertension | 23 | 44738 | 74 | 144650 | 1.00 | 1 (0.63–1.6) |
| Renovascular disorders | 7 | 44754 | 28 | 144696 | 0.81 | 0.81 (0.35–1.85) |
| Rhabdomyolysis/myopathy | 9 | 44752 | 47 | 144677 | 0.62 | 0.62 (0.3–1.26) |
| Scleral disorders | 8 | 44753 | 26 | 144698 | 0.99 | 0.99 (0.45–2.2) |
| Sepsis | 378 | 44383 | 955 | 143769 | 1.28 | 1.28 (1.14–1.45) |
| Severe cutaneous adverse reactions | 190 | 44571 | 309 | 144415 | 1.99 | 1.99 (1.66–2.39) |
| Skin neoplasms, malignant and unspecified | 351 | 44410 | 1512 | 143212 | 0.75 | 0.75 (0.67–0.84) |
| Systemic lupus erythematosus | 227 | 44534 | 779 | 143945 | 0.94 | 0.94 (0.81–1.09) |
| Thrombophlebitis | 12 | 44749 | 36 | 144688 | 1.08 | 1.08 (0.56–2.07) |
| Tubulointerstitial diseases | 7 | 44754 | 40 | 144684 | 0.57 | 0.57 (0.25–1.26) |
| Uterine and fallopian tube neoplasms, malignant and unspecified | 49 | 44712 | 183 | 144541 | 0.87 | 0.87 (0.63–1.19) |
| Vasculitis | 114 | 44647 | 221 | 144503 | 1.67 | 1.67 (1.33–2.09) |

AE, adverse event; ROR, reporting odds ratio; PRR, proportional reporting ratio; CI, confidence interval.

**Supplementary Table S5.** Disproportionality analysis of adverse events at the standardised MedDRA query level reported in patients with psoriasis or related disorders receiving secukinumab or non-biologics

| **Standardised MedDRA Query** | **With secukinumab** | | **With non-biologics** | | **Disproportionality statistic** | |
| --- | --- | --- | --- | --- | --- | --- |
|  | **Number of AEs of interest** | **Number of other AEs** | **Number of AEs of interest** | **Number of other AEs** | **PRR** | **ROR (95% CI)** |
| Acute renal failure | 321 | 44440 | 339 | 66666 | 1.42 | 1.42 (1.22–1.66) |
| Agranulocytosis | 40 | 44721 | 280 | 66725 | 0.21 | 0.21 (0.15–0.3) |
| Anaphylactic reactions | 129 | 44632 | 52 | 66953 | **3.71** | **3.72 (2.7–5.14)** |
| Angioedema | 1209 | 43552 | 589 | 66416 | **3.07** | **3.13 (2.83–3.46)** |
| Arthritis | 2164 | 42597 | 712 | 66293 | **4.55** | **4.73 (4.34–5.15)** |
| Biliary disorders | 275 | 44486 | 274 | 66731 | 1.50 | 1.51 (1.27–1.78) |
| Breast neoplasms, malignant and unspecified | 226 | 44535 | 153 | 66852 | **2.21** | **2.22 (1.81–2.72)** |
| Cardiac arrhythmias | 303 | 44458 | 315 | 66690 | 1.44 | 1.44 (1.23–1.69) |
| Cardiac failure | 248 | 44513 | 195 | 66810 | 1.90 | 1.91 (1.58–2.3) |
| Cardiomyopathy | 50 | 44711 | 13 | 66992 | **5.76** | **5.76 (3.13–10.61)** |
| Central nervous system vascular disorders | 471 | 44290 | 320 | 66685 | **2.20** | **2.22 (1.92–2.56)** |
| Chronic kidney disease | 166 | 44595 | 166 | 66839 | 1.50 | 1.5 (1.21–1.86) |
| Conjunctival disorders | 329 | 44432 | 181 | 66824 | **2.72** | **2.73 (2.28–3.28)** |
| Demyelination | 116 | 44645 | 70 | 66935 | **2.48** | **2.48 (1.85–3.34)** |
| Depression and suicide/self–injury | 761 | 44000 | 2726 | 64279 | 0.42 | 0.41 (0.38–0.44) |
| Drug reaction with eosinophilia and systemic symptoms syndrome | 5 | 44756 | 7 | 66998 | 1.07 | 1.07 (0.34–3.37) |
| Dyslipidaemia | 245 | 44516 | 119 | 66886 | **3.08** | **3.09 (2.48–3.85)** |
| Embolic and thrombotic events | 1215 | 43546 | 714 | 66291 | **2.55** | **2.59 (2.36–2.84)** |
| Extravasation events (injections, infusions and implants) | 65 | 44696 | 4 | 67001 | **24.33** | **24.36 (8.87–66.87)** |
| Gastrointestinal nonspecific inflammation and dysfunctional conditions | 5133 | 39628 | 24203 | 42802 | 0.32 | 0.23 (0.22–0.24) |
| Gastrointestinal perforation, ulceration, haemorrhage or obstruction | 1437 | 43324 | 935 | 66070 | **2.30** | **2.34 (2.16–2.55)** |
| Guillain–Barre syndrome | 38 | 44723 | 10 | 66995 | **5.69** | **5.69 (2.84–11.43)** |
| Haematopoietic cytopenias | 400 | 44361 | 570 | 66435 | 1.05 | 1.05 (0.92–1.19) |
| Haemolytic disorders | 27 | 44734 | 6 | 66999 | **6.74** | 6.74 (2.78–16.33) |
| Haemorrhages | 3073 | 41688 | 1203 | 65802 | **3.82** | 4.03 (3.77–4.31) |
| Hypersensitivity | 5038 | 39723 | 4019 | 62986 | 1.88 | 1.99 (1.9–2.08) |
| Hypertension | 1067 | 43694 | 668 | 66337 | **2.39** | **2.43 (2.2–2.67)** |
| Hepatic disorders | 1382 | 43379 | 1039 | 65966 | 1.99 | **2.02 (1.86–2.19)** |
| Immune-mediated/autoimmune disorders | 10963 | 33798 | 13448 | 53557 | 1.22 | 1.29 (1.26–1.33) |
| Infective Pneumonia | 1051 | 43710 | 703 | 66302 | **2.24** | **2.27 (2.06–2.5)** |
| Ischaemic colitis | 55 | 44706 | 8 | 66997 | **10.29** | **10.3 (4.91–21.63)** |
| Ischaemic heart disease | 509 | 44252 | 290 | 66715 | **2.63** | **2.65 (2.29–3.06)** |
| Lipodystrophy | 5 | 44756 | 2 | 67003 | **3.74** | **3.74 (0.73–19.29)** |
| Malignancies | 1805 | 42956 | 1403 | 65602 | 1.93 | 1.96 (1.83–2.11) |
| Malignant lymphomas | 155 | 44606 | 124 | 66881 | 1.87 | 1.87 (1.48–2.37) |
| Myelodysplastic syndrome | 11 | 44750 | 16 | 66989 | 1.03 | 1.03 (0.48–2.22) |
| Ocular infections | 232 | 44529 | 74 | 66931 | **4.69** | **4.71 (3.63–6.12)** |
| Oropharyngeal disorders | 343 | 44418 | 1259 | 65746 | 0.41 | 0.4 (0.36–0.45) |
| Opportunistic infections | 374 | 44387 | 185 | 66820 | **3.03** | **3.04 (2.55–3.63)** |
| Ovarian neoplasms, malignant and unspecified | 21 | 44740 | 16 | 66989 | 1.96 | 1.97 (1.03–3.77) |
| Peripheral neuropathy | 299 | 44462 | 177 | 66828 | **2.53** | **2.54 (2.11–3.06)** |
| Premalignant disorders | 615 | 44146 | 204 | 66801 | **4.51** | **4.56 (3.89–5.35)** |
| Prostate neoplasms, malignant and unspecified | 100 | 44661 | 74 | 66931 | **2.02** | **2.03 (1.5–2.74)** |
| Pseudomembranous colitis | 71 | 44690 | 51 | 66954 | **2.08** | **2.09 (1.46–2.99)** |
| Pulmonary hypertension | 23 | 44738 | 17 | 66988 | **2.03** | **2.03 (1.08–3.79)** |
| Renovascular disorders | 7 | 44754 | 5 | 67000 | **2.10** | **2.1 (0.67–6.6)** |
| Rhabdomyolysis/myopathy | 9 | 44752 | 16 | 66989 | 0.84 | 0.84 (0.37–1.91) |
| Scleral disorders | 8 | 44753 | 3 | 67002 | **3.99** | **3.99 (1.06–15.05)** |
| Sepsis | 378 | 44383 | 257 | 66748 | **2.20** | **2.21 (1.89–2.59)** |
| Severe cutaneous adverse reactions | 190 | 44571 | 192 | 66813 | 1.48 | 1.48 (1.21–1.81) |
| Skin neoplasms, malignant and unspecified | 351 | 44410 | 268 | 66737 | 1.96 | 1.97 (1.68–2.31) |
| Systemic lupus erythematosus | 227 | 44534 | 59 | 66946 | **5.76** | **5.78 (4.34–7.7)** |
| Thrombophlebitis | 12 | 44749 | 5 | 67000 | **3.59** | **3.59 (1.27–10.2)** |
| Tubulointerstitial diseases | 7 | 44754 | 23 | 66982 | 0.46 | 0.46 (0.2–1.06) |
| Uterine and fallopian tube neoplasms, malignant and unspecified | 49 | 44712 | 32 | 66973 | **2.29** | **2.29 (1.47–3.58)** |
| Vasculitis | 114 | 44647 | 47 | 66958 | **3.63** | **3.64 (2.59–5.11)** |

AE, adverse event; ROR, reporting odds ratio; PRR, proportional reporting ratio; CI, confidence interval.

**Supplementary Table S6.** Disproportionality analysis of adverse events at the preferred term level reported in patient with psoriasis or related disorders receiving secukinumab or biologics or non-biologics

| **Preferred Terms*** | **Secukinumab versus other biologics** | | **Secukinumab versus non-biologics** | |
| --- | --- | --- | --- | --- |
|  | **PRR** | **ROR (95% CI)** | **PRR** | **ROR (95% CI)** |
| Abdominal discomfort | **2.88** | **2.92 (2.65–3.21)** | 0.35 | 0.34 (0.32-0.37) |
| Abdominal distension | 1.64 | 1.64 (1.38–1.96) | 0.82 | 0.82 (0.68-0.98) |
| Abdominal pain | **2.03** | **2.04 (1.85–2.26)** | 1.18 | 1.19 (1.07-1.32) |
| Abdominal pain upper | **2.15** | **2.16 (1.95–2.4)** | 0.47 | 0.46 (0.42-0.5) |
| Abscess limb | **2.94** | **2.94 (2.3–3.77)** | **8.98** | **9 (5.61-14.46)** |
| Accident | 1.41 | 1.41 (1.12–1.79) | **6.30** | **6.31 (4.04-9.85)** |
| Accidental exposure to product | 0.63 | 0.63 (0.56–0.71) | **48.20** | **48.54 (25.87-91.1)** |
| Acne | **2.05** | **2.06 (1.72–2.46)** | **4.02** | **4.03 (3.08-5.28)** |
| Acute myocardial infarction | 1.47 | 1.48 (1.18–1.84) | **4.74** | **4.75 (3.26-6.91)** |
| Adverse drug reaction | 0.60 | 0.6 (0.5–0.73) | 0.25 | 0.25 (0.2-0.3) |
| Ageusia | **6.83** | **6.87 (5.63–8.39)** | **5.22** | **5.25 (4.13-6.67)** |
| Alanine aminotransferase increased | 1.28 | 1.28 (1.05–1.56) | **3.46** | **3.47 (2.55-4.72)** |
| Alopecia | 1.50 | 1.5 (1.36–1.66) | 1.47 | 1.48 (1.32-1.66) |
| Amnesia | 0.99 | 0.99 (0.85–1.15) | **2.17** | **2.18 (1.76-2.69)** |
| Anaemia | 1.18 | 1.18 (1–1.4) | **2.22** | **2.23 (1.78-2.79)** |
| Anaphylactic reaction | 1.00 | 1 (0.8–1.24) | **4.25** | **4.26 (2.94-6.17)** |
| Angina pectoris | **2.24** | **2.25 (1.86–2.71)** | **4.35** | **4.36 (3.28-5.8)** |
| Angioedema | **2.48** | **2.49 (2.12–2.92)** | **4.59** | **4.62 (3.62-5.88)** |
| Ankle fracture | 0.85 | 0.85 (0.69–1.05) | **2.57** | **2.58 (1.89-3.51)** |
| Ankylosing spondylitis | **3.50** | **3.52 (3–4.12)** | **21.50** | **21.65 (14.05-33.36)** |
| Anosmia | **10.51** | **10.56 (8.11–13.75)** | **9.73** | **9.78 (6.88-13.89)** |
| Anxiety | 1.36 | 1.37 (1.23–1.51) | 1.17 | 1.17 (1.04-1.31) |
| Aphasia | 1.69 | 1.69 (1.35–2.13) | **8.38** | **8.4 (5.22-13.52)** |
| Aphonia | 1.58 | 1.58 (1.32–1.89) | **2.90** | **2.9 (2.26-3.73)** |
| Aphthous ulcer | **7.31** | **7.32 (5.18–10.37)** | **7.08** | **7.09 (4.48-11.23)** |
| Arthralgia | 1.76 | 1.84 (1.77–1.91) | **3.11** | **3.32 (3.15-3.51)** |
| Arthritis | 1.75 | 1.77 (1.65–1.91) | **4.47** | **4.55 (4.05-5.13)** |
| Arthritis infective | 1.55 | 1.55 (1.23–1.95) | **4.49** | **4.5 (3.08-6.56)** |
| Arthropathy | 1.47 | 1.48 (1.33–1.65) | 1.75 | 1.76 (1.54-2.01) |
| Ascites | **2.26** | **2.27 (1.84–2.8)** | **5.64** | **5.66 (3.97-8.05)** |
| Aspartate aminotransferase increased | 1.36 | 1.36 (1.09–1.69) | **7.76** | **7.77 (4.92-12.27)** |
| Asthenia | 1.54 | 1.54 (1.42–1.68) | 1.92 | 1.94 (1.74-2.16) |
| Asthma | 1.50 | 1.51 (1.35–1.68) | **3.43** | **3.45 (2.92-4.08)** |
| Atrial fibrillation | 0.88 | 0.88 (0.73–1.06) | 1.49 | 1.49 (1.18-1.88) |
| Back pain | 1.52 | 1.53 (1.42–1.65) | 1.90 | 1.92 (1.76-2.1) |
| Bacterial infection | 1.57 | 1.57 (1.25–1.97) | **3.50** | **3.51 (2.49-4.94)** |
| Balance disorder | 0.85 | 0.85 (0.72–1.01) | 1.97 | 1.98 (1.58-2.48) |
| Basal cell carcinoma | 0.64 | 0.63 (0.55–0.73) | **4.24** | **4.26 (3.32-5.46)** |
| Behcet's syndrome | **5.75** | **5.76 (4.23–7.84)** | **2.99** | **3 (2.18-4.13)** |
| Blister | **2.99** | **3.04 (2.79–3.31)** | **5.68** | **5.79 (5.06-6.63)** |
| Blood cholesterol increased | 0.73 | 0.73 (0.59–0.91) | **3.14** | **3.15 (2.25-4.39)** |
| Blood creatinine increased | 1.77 | 1.78 (1.42–2.22) | 1.80 | 1.8 (1.38-2.36) |
| Blood glucose increased | 1.20 | 1.21 (1.03–1.41) | **2.05** | **2.05 (1.67-2.53)** |
| Blood pressure decreased | **2.09** | **2.09 (1.65–2.66)** | **2.99** | **3 (2.17-4.15)** |
| Blood pressure increased | 1.76 | 1.76 (1.56–1.99) | 1.86 | 1.86 (1.61-2.16) |
| Blood pressure systolic increased | **11.71** | **11.73 (7.78–17.7)** | **8.27** | **8.29 (5.08-13.52)** |
| Bone pain | **2.45** | **2.46 (2.06–2.93)** | **2.82** | **2.83 (2.26-3.53)** |
| Breast cancer | 1.23 | 1.23 (1.08–1.41) | 1.95 | 1.96 (1.65-2.32) |
| Bronchitis | 1.24 | 1.25 (1.17–1.34) | **2.61** | **2.66 (2.42-2.92)** |
| Burning sensation | **2.23** | **2.25 (2.05–2.47)** | **3.56** | **3.61 (3.16-4.12)** |
| Bursitis | 1.70 | 1.7 (1.44–2.01) | **7.86** | **7.89 (5.63-11.07)** |
| Candida infection | **4.20** | **4.22 (3.52–5.06)** | **12.58** | **12.65 (8.77-18.26)** |
| Cardiac disorder | 0.96 | 0.96 (0.81–1.14) | 1.31 | 1.31 (1.07-1.61) |
| Cataract | 0.58 | 0.58 (0.47–0.72) | 1.86 | 1.86 (1.4-2.48) |
| Cellulitis | 1.62 | 1.64 (1.53–1.76) | **5.26** | **5.38 (4.76-6.08)** |
| Cerebrovascular accident | 0.97 | 0.97 (0.88–1.07) | **2.24** | **2.25 (1.98-2.57)** |
| Chest discomfort | 1.74 | 1.75 (1.61–1.91) | **2.94** | **2.98 (2.65-3.35)** |
| Chest pain | 1.30 | 1.3 (1.22–1.39) | **2.43** | **2.47 (2.26-2.71)** |
| Chills | 1.76 | 1.77 (1.63–1.93) | **3.31** | **3.36 (2.96-3.8)** |
| Chronic obstructive pulmonary disease | 0.77 | 0.77 (0.63–0.93) | **2.04** | **2.04 (1.56-2.68)** |
| Colitis | **2.42** | **2.42 (2.02–2.91)** | **6.02** | **6.04 (4.43-8.24)** |
| Colitis ulcerative | **2.72** | **2.73 (2.37–3.14)** | **10.31** | **10.38 (7.76-13.89)** |
| Concomitant disease aggravated | **209.35** | **210.57 (78.43–565.32)** | NA | NA |
| Condition aggravated | 0.97 | 0.97 (0.89–1.05) | **2.86** | **2.89 (2.55-3.27)** |
| Confusional state | 1.36 | 1.37 (1.2–1.55) | **2.26** | **2.27 (1.92-2.69)** |
| Conjunctivitis | **3.11** | **3.12 (2.63–3.71)** | **7.92** | **7.96 (5.85-10.84)** |
| Constipation | 1.49 | 1.49 (1.27–1.75) | 0.91 | 0.91 (0.77-1.08) |
| Contraindicated product administered | **2.45** | **2.45 (2.04–2.95)** | **11.63** | **11.68 (7.76-17.57)** |
| Contusion | 1.39 | 1.4 (1.32–1.49) | **4.37** | **4.5 (4.09-4.95)** |
| Coronavirus infection | **3.96** | **3.97 (3.16–4.99)** | **3.84** | **3.85 (2.88-5.14)** |
| Cough | **2.21** | **2.26 (2.12–2.41)** | **2.75** | **2.82 (2.6-3.06)** |
| COVID-19 | **2.69** | **2.76 (2.59–2.94)** | **4.84** | **5.01 (4.55-5.51)** |
| C-reactive protein increased | **4.95** | **4.99 (4.27–5.83)** | **8.38** | **8.45 (6.57-10.86)** |
| Crohn's disease | 1.56 | 1.56 (1.39–1.76) | **8.00** | **8.06 (6.31-10.3)** |
| Crying | **2.20** | **2.21 (1.94–2.53)** | 1.33 | 1.34 (1.16-1.54) |
| Cyst | 0.82 | 0.82 (0.69–0.98) | **3.38** | **3.39 (2.56-4.49)** |
| Cystitis | 1.64 | 1.64 (1.47–1.84) | **3.71** | **3.74 (3.16-4.43)** |
| Death | 1.13 | 1.13 (1.02–1.25) | 1.67 | 1.68 (1.48-1.9) |
| Decreased appetite | 1.57 | 1.58 (1.44–1.73) | 0.39 | 0.38 (0.35-0.41) |
| Decreased immune responsiveness | **6.02** | **6.06 (5.1–7.21)** | **22.89** | **23.07 (15.26-34.87)** |
| Dehydration | 0.94 | 0.94 (0.8–1.12) | 1.33 | 1.33 (1.08-1.64) |
| Depressed mood | **2.21** | **2.21 (1.83–2.67)** | 1.09 | 1.1 (0.91-1.32) |
| Depression | 1.14 | 1.14 (1.03–1.27) | 0.32 | 0.31 (0.28-0.34) |
| Dermatitis | 1.94 | 1.94 (1.51–2.48) | **4.28** | **4.28 (2.91-6.3)** |
| Dermatitis contact | 1.20 | 1.2 (0.99–1.46) | **3.62** | **3.63 (2.67-4.95)** |
| Device issue | 0.23 | 0.22 (0.2–0.25) | **50.73** | **51.07 (26.32-99.11)** |
| Device malfunction | **2.09** | **2.1 (1.87–2.36)** | **87.38** | **88.3 (43.89-177.62)** |
| Diabetes mellitus | 1.35 | 1.35 (1.24–1.48) | **4.09** | **4.14 (3.6-4.76)** |
| Diarrhoea | **3.00** | **3.1 (2.93–3.29)** | 0.27 | 0.23 (0.22-0.24) |
| Diarrhoea haemorrhagic | **4.25** | **4.26 (3.29–5.51)** | **3.04** | **3.05 (2.27-4.09)** |
| Discomfort | **2.19** | **2.2 (1.93–2.51)** | 1.76 | 1.77 (1.52-2.06) |
| Diverticulitis | 1.28 | 1.29 (1.15–1.44) | **3.01** | **3.03 (2.57-3.57)** |
| Dizziness | 1.22 | 1.23 (1.17–1.29) | 1.03 | 1.03 (0.98-1.09) |
| Drug eruption | **2.28** | **2.29 (1.96–2.67)** | **7.17** | **7.21 (5.42-9.59)** |
| Drug hypersensitivity | 1.31 | 1.32 (1.17–1.48) | **3.91** | **3.93 (3.24-4.77)** |
| Drug ineffective | **2.18** | **2.39 (2.31–2.47)** | **2.65** | **2.94 (2.82-3.07)** |
| Drug intolerance | **3.18** | **3.2 (2.82–3.64)** | **3.10** | **3.12 (2.66-3.66)** |
| Dry mouth | 1.97 | 1.98 (1.62–2.41) | **2.09** | **2.1 (1.65-2.67)** |
| Dry skin | **3.15** | **3.18 (2.86–3.54)** | **3.45** | **3.49 (3.04-4.01)** |
| Dry throat | **2.42** | **2.43 (1.89–3.12)** | **4.76** | **4.76 (3.24-7.01)** |
| Dysgeusia | 1.56 | 1.56 (1.31–1.86) | 0.94 | 0.94 (0.78-1.12) |
| Dyspepsia | 1.66 | 1.66 (1.39–1.98) | 0.36 | 0.35 (0.3-0.41) |
| Dysphagia | **2.54** | **2.54 (2.14–3.02)** | **4.36** | **4.38 (3.4-5.64)** |
| Dysphonia | 1.88 | 1.89 (1.71–2.09) | **3.77** | **3.81 (3.27-4.43)** |
| Dyspnoea | 1.86 | 1.91 (1.82–2.01) | **3.12** | **3.24 (3.03-3.48)** |
| Dysstasia | **2.54** | **2.56 (2.28–2.87)** | **5.85** | **5.91 (4.88-7.16)** |
| Dysuria | 1.95 | 1.95 (1.58–2.4) | **4.62** | **4.63 (3.32-6.46)** |
| Ear infection | 1.70 | 1.71 (1.56–1.87) | **3.55** | **3.6 (3.15-4.11)** |
| Ear pain | 1.83 | 1.84 (1.5–2.25) | **4.43** | **4.44 (3.22-6.12)** |
| Eating disorder | **5.31** | **5.33 (4.21–6.74)** | **3.36** | **3.37 (2.6-4.37)** |
| Eczema | **2.73** | **2.74 (2.26–3.32)** | **3.79** | **3.8 (2.91-4.97)** |
| Emotional distress | 1.25 | 1.25 (0.99–1.58) | **6.30** | **6.31 (4.04-9.85)** |
| Enthesopathy | **2.84** | **2.84 (2.26–3.58)** | **10.18** | **10.21 (6.38-16.32)** |
| Epistaxis | 0.77 | 0.77 (0.65–0.92) | 1.11 | 1.11 (0.9-1.36) |
| Erysipelas | **3.26** | **3.27 (2.75–3.89)** | **12.26** | **12.32 (8.54-17.79)** |
| Erythema | **2.15** | **2.18 (2.03–2.35)** | **4.87** | **4.98 (4.42-5.6)** |
| Exposure during pregnancy | 0.35 | 0.35 (0.3–0.42) | **7.29** | **7.31 (4.93-10.82)** |
| Eye infection | **2.16** | **2.17 (1.85–2.55)** | **5.06** | **5.08 (3.92-6.59)** |
| Eye swelling | 1.89 | 1.89 (1.52–2.35) | **3.48** | **3.49 (2.54-4.79)** |
| Fall | 0.89 | 0.88 (0.81–0.96) | **2.35** | **2.38 (2.12-2.67)** |
| Fatigue | 1.57 | 1.6 (1.52–1.68) | 1.74 | 1.78 (1.68-1.89) |
| Feeding disorder | **2.42** | **2.43 (1.89–3.12)** | 1.19 | 1.19 (0.92-1.53) |
| Feeling abnormal | 1.75 | 1.77 (1.62–1.92) | 1.81 | 1.83 (1.65-2.03) |
| Feeling hot | 1.17 | 1.17 (0.98–1.39) | **3.56** | **3.57 (2.72-4.69)** |
| Fibromyalgia | 1.86 | 1.87 (1.56–2.24) | **4.69** | **4.71 (3.52-6.29)** |
| Flatulence | 1.97 | 1.98 (1.55–2.51) | 0.38 | 0.37 (0.3-0.46) |
| Fluid retention | 1.33 | 1.33 (1.13–1.58) | **2.71** | **2.71 (2.13-3.45)** |
| Flushing | 1.25 | 1.26 (1.09–1.45) | 1.95 | 1.96 (1.63-2.35) |
| Folliculitis | **3.05** | **3.06 (2.47–3.78)** | **4.97** | **4.98 (3.63-6.84)** |
| Food poisoning | 1.50 | 1.5 (1.2–1.87) | 1.90 | 1.9 (1.44-2.5) |
| Foot fracture | 1.10 | 1.1 (0.92–1.31) | 1.94 | 1.94 (1.54-2.45) |
| Fungal infection | 1.95 | 1.95 (1.69–2.25) | **4.52** | **4.54 (3.63-5.69)** |
| Furuncle | 1.54 | 1.54 (1.31–1.81) | **5.31** | **5.33 (4.02-7.07)** |
| Gait disturbance | 1.52 | 1.56 (1.49–1.63) | **4.64** | **4.87 (4.5-5.26)** |
| Gait inability | 1.64 | 1.65 (1.49–1.82) | **4.88** | **4.93 (4.18-5.82)** |
| Gastric disorder | 1.08 | 1.08 (0.87–1.35) | 0.22 | 0.22 (0.18-0.27) |
| Gastroenteritis | **2.63** | **2.63 (2.12–3.27)** | **4.82** | **4.83 (3.47-6.72)** |
| Gastroenteritis viral | 1.41 | 1.41 (1.2–1.66) | 1.28 | 1.28 (1.07-1.54) |
| Gastrointestinal disorder | **2.04** | **2.05 (1.76–2.39)** | 0.54 | 0.54 (0.47-0.62) |
| Gastrointestinal infection | 1.56 | 1.56 (1.24–1.96) | **3.58** | **3.59 (2.54-5.06)** |
| Gastrooesophageal reflux disease | 1.31 | 1.31 (1.08–1.59) | 0.47 | 0.47 (0.39-0.57) |
| General physical health deterioration | **2.50** | **2.51 (2.18–2.91)** | **4.36** | **4.38 (3.53-5.43)** |
| Glossodynia | **2.76** | **2.76 (2.26–3.39)** | **21.71** | **21.79 (12.14-39.11)** |
| Gout | 1.26 | 1.26 (1.06–1.5) | 1.53 | 1.53 (1.25-1.88) |
| Grip strength decreased | **2.18** | **2.18 (1.74–2.74)** | **12.37** | **12.41 (7.26-21.2)** |
| Haematochezia | **2.62** | **2.63 (2.3–3.01)** | **2.35** | **2.36 (2.01-2.77)** |
| Haematuria | **2.04** | **2.04 (1.69–2.47)** | **2.80** | **2.81 (2.18-3.61)** |
| Haemoptysis | 1.46 | 1.47 (1.2–1.79) | 1.84 | 1.85 (1.45-2.36) |
| Haemorrhage | 1.62 | 1.62 (1.36–1.93) | **3.31** | **3.32 (2.57-4.29)** |
| Haemorrhoids | 1.49 | 1.49 (1.2–1.84) | **2.05** | **2.05 (1.57-2.69)** |
| Hand deformity | 1.72 | 1.72 (1.39–2.13) | **12.35** | **12.38 (7.37-20.81)** |
| Headache | 1.25 | 1.26 (1.19–1.33) | 0.30 | 0.27 (0.26-0.29) |
| Heart rate increased | 2.00 | 2 (1.67–2.4) | 1.41 | 1.42 (1.16-1.73) |
| Hepatic cirrhosis | 0.84 | 0.84 (0.68–1.05) | **2.39** | **2.39 (1.75-3.27)** |
| Hepatic enzyme increased | 0.96 | 0.95 (0.83–1.09) | **2.87** | **2.88 (2.36-3.52)** |
| Hepatic steatosis | 0.90 | 0.89 (0.77–1.03) | **2.76** | **2.77 (2.23-3.44)** |
| Herpes zoster | 1.00 | 1 (0.93–1.08) | **2.32** | **2.34 (2.11-2.6)** |
| Hip fracture | 1.22 | 1.22 (0.98–1.5) | **2.32** | **2.33 (1.74-3.11)** |
| Hot flush | 1.64 | 1.65 (1.47–1.84) | 1.63 | 1.63 (1.43-1.87) |
| Hypercalcaemia | **2.55** | **2.55 (1.97–3.3)** | **3.54** | **3.54 (2.49-5.04)** |
| Hyperhidrosis | 1.27 | 1.28 (1.15–1.41) | 1.85 | 1.86 (1.64-2.12) |
| Hypersensitivity | 1.94 | 1.96 (1.8–2.12) | **5.46** | **5.56 (4.84-6.38)** |
| Hypersensitivity vasculitis | 1.65 | 1.65 (1.31–2.07) | **20.96** | **21.01 (10.25-43.05)** |
| Hypersomnia | 1.36 | 1.36 (1.1–1.68) | 1.60 | 1.61 (1.24-2.08) |
| Hypertension | 1.28 | 1.28 (1.16–1.42) | **2.74** | **2.77 (2.4-3.19)** |
| Hypoacusis | 1.08 | 1.08 (0.88–1.33) | **2.37** | **2.37 (1.77-3.17)** |
| Hypoaesthesia | 0.96 | 0.96 (0.89–1.04) | **3.17** | **3.21 (2.85-3.63)** |
| Hypokinesia | **8.44** | **8.47 (6.27–11.43)** | **14.41** | **14.45 (8.64-24.19)** |
| Hypotension | 1.05 | 1.05 (0.87–1.28) | 1.79 | 1.79 (1.39-2.31) |
| Ill-defined disorder | **2.47** | **2.48 (2.08–2.95)** | **6.15** | **6.18 (4.6-8.3)** |
| Illness | **3.75** | **3.78 (3.27–4.36)** | **3.89** | **3.91 (3.25-4.71)** |
| Immune system disorder | 1.51 | 1.52 (1.23–1.86) | **2.26** | **2.27 (1.73-2.97)** |
| Immunodeficiency | 0.87 | 0.87 (0.71–1.06) | **5.84** | **5.86 (3.95-8.69)** |
| Impaired healing | 0.78 | 0.78 (0.65–0.93) | **3.52** | **3.52 (2.62-4.73)** |
| Inappropriate schedule of product administration | **3.76** | **3.98 (3.78–4.19)** | **20.21** | **21.73 (19.07-24.77)** |
| Incorrect dose administered | 1.47 | 1.49 (1.4–1.58) | **7.79** | **8.03 (7.1-9.09)** |
| Incorrect dose administered by device | **2.16** | **2.16 (1.85–2.52)** | NA | NA |
| Incorrect route of product administration | **7.08** | **7.13 (5.98–8.52)** | **36.68** | **36.99 (22.43-60.99)** |
| Infection | 1.48 | 1.49 (1.37–1.61) | **2.97** | **3.01 (2.69-3.37)** |
| Inflammation | **2.80** | **2.82 (2.55–3.12)** | **8.03** | **8.14 (6.76-9.81)** |
| Inflammatory bowel disease | **5.73** | **5.74 (4.25–7.77)** | **15.92** | **15.96 (8.6-29.62)** |
| Influenza | 1.81 | 1.86 (1.76–1.96) | **3.12** | **3.23 (3-3.48)** |
| Influenza like illness | 1.80 | 1.81 (1.6–2.04) | **2.66** | **2.68 (2.28-3.15)** |
| Infusion related reaction | 0.71 | 0.71 (0.64–0.8) | **26.09** | **26.3 (16.94-40.83)** |
| Injection site bruising | 0.95 | 0.95 (0.91–0.98) | **201.34** | **216.91 (145.14-324.16)** |
| Injection site discolouration | 0.30 | 0.3 (0.25–0.36) | NA | NA |
| Injection site discomfort | 0.45 | 0.45 (0.37–0.55) | NA | NA |
| Injection site erythema | 0.10 | 0.09 (0.08–0.1) | **47.30** | **47.8 (28.58-79.93)** |
| Injection site extravasation | 0.62 | 0.62 (0.51–0.75) | **22.45** | **22.51 (11-46.05)** |
| Injection site haemorrhage | 0.49 | 0.48 (0.45–0.5) | **168.41** | **173.61 (98.34-306.52)** |
| Injection site mass | 0.53 | 0.52 (0.46–0.6) | NA | NA |
| Injection site pain | 0.37 | 0.33 (0.32–0.35) | **73.23** | **77.22 (58.01-102.8)** |
| Injection site pruritus | 0.17 | 0.16 (0.15–0.18) | **208.08** | **210.02 (67.46-653.9)** |
| Injection site rash | 0.19 | 0.19 (0.16–0.22) | **42.66** | **42.82 (18.97-96.66)** |
| Injection site reaction | 0.23 | 0.22 (0.2–0.25) | **17.32** | **17.44 (11.85-25.66)** |
| Injection site swelling | 0.18 | 0.17 (0.16–0.19) | **50.90** | **51.28 (27.34-96.18)** |
| Injection site urticaria | 0.17 | 0.17 (0.14–0.19) | **38.92** | **39.1 (19.29-79.23)** |
| Injury | **2.50** | **2.51 (2.06–3.04)** | **8.47** | **8.5 (5.83-12.38)** |
| Insomnia | 1.71 | 1.73 (1.61–1.86) | 0.67 | 0.66 (0.62-0.71) |
| Interstitial lung disease | 1.08 | 1.08 (0.89–1.32) | **2.28** | **2.28 (1.74-2.99)** |
| Irritability | 1.12 | 1.12 (0.9–1.39) | 0.39 | 0.39 (0.32-0.48) |
| Irritable bowel syndrome | **2.81** | **2.82 (2.42–3.28)** | **2.17** | **2.18 (1.83-2.59)** |
| Joint dislocation | 0.89 | 0.89 (0.73–1.08) | **8.85** | **8.87 (5.64-13.94)** |
| Joint injury | 1.42 | 1.43 (1.25–1.63) | **3.81** | **3.83 (3.12-4.7)** |
| Joint stiffness | 1.31 | 1.32 (1.15–1.5) | **4.06** | **4.09 (3.3-5.06)** |
| Joint swelling | 1.83 | 1.85 (1.72–1.99) | **4.87** | **4.98 (4.42-5.6)** |
| Kidney infection | 1.31 | 1.31 (1.16–1.48) | **4.74** | **4.78 (3.88-5.88)** |
| Laryngitis | 1.94 | 1.94 (1.61–2.35) | **3.95** | **3.96 (2.98-5.26)** |
| Lethargy | 1.83 | 1.84 (1.63–2.07) | 1.88 | 1.89 (1.64-2.18) |
| Ligament sprain | 1.11 | 1.11 (0.91–1.35) | **4.30** | **4.31 (3.11-5.99)** |
| Limb discomfort | **3.77** | **3.79 (3.18–4.52)** | **4.57** | **4.6 (3.62-5.84)** |
| Limb injury | 1.23 | 1.23 (1.04–1.45) | **3.84** | **3.85 (2.96-5.02)** |
| Lip swelling | **2.23** | **2.24 (1.94–2.58)** | **3.91** | **3.93 (3.19-4.84)** |
| Liver disorder | 1.11 | 1.11 (0.93–1.34) | **2.18** | **2.19 (1.71-2.8)** |
| Liver function test increased | 1.66 | 1.66 (1.31–2.11) | **2.16** | **2.16 (1.59-2.93)** |
| Liver injury | **4.62** | **4.64 (3.79–5.69)** | **5.46** | **5.48 (4.14-7.26)** |
| Localised infection | 1.47 | 1.47 (1.27–1.7) | **4.54** | **4.56 (3.58-5.83)** |
| Loss of consciousness | 0.57 | 0.57 (0.47–0.67) | 1.96 | 1.96 (1.53-2.52) |
| Lower limb fracture | 1.40 | 1.4 (1.15–1.71) | **2.87** | **2.88 (2.17-3.81)** |
| Lower respiratory tract infection | 1.36 | 1.37 (1.28–1.47) | **13.17** | **13.51 (11.32-16.12)** |
| Lung disorder | 1.23 | 1.23 (1–1.5) | **2.65** | **2.65 (1.99-3.53)** |
| Lung neoplasm malignant | 0.92 | 0.92 (0.77–1.1) | **2.17** | **2.17 (1.7-2.76)** |
| Lupus-like syndrome | 0.64 | 0.64 (0.53–0.77) | **6.59** | **6.6 (4.44-9.82)** |
| Lymphadenopathy | 1.09 | 1.09 (0.92–1.29) | **4.35** | **4.37 (3.29-5.79)** |
| Macule | **15.87** | **15.99 (12.49–20.47)** | **31.02** | **31.27 (19.48-50.19)** |
| Malaise | **2.33** | **2.4 (2.28–2.53)** | **3.00** | **3.12 (2.91-3.34)** |
| Malignant melanoma | 0.69 | 0.69 (0.57–0.83) | 1.99 | 1.99 (1.53-2.59) |
| Mass | 1.31 | 1.31 (1.05–1.63) | **5.19** | **5.2 (3.51-7.71)** |
| Maternal exposure during pregnancy | 1.72 | 1.73 (1.57–1.9) | **13.73** | **13.91 (10.87-17.81)** |
| Memory impairment | 1.28 | 1.28 (1.17–1.4) | 1.01 | 1.02 (0.92-1.12) |
| Migraine | 1.31 | 1.31 (1.19–1.45) | 0.52 | 0.51 (0.46-0.56) |
| Mobility decreased | 0.95 | 0.95 (0.89–1.01) | **13.00** | **13.35 (11.23-15.87)** |
| Mouth ulceration | **4.26** | **4.27 (3.29–5.53)** | **2.10** | **2.1 (1.61-2.73)** |
| Movement disorder | **13.48** | **13.54 (10.03–18.28)** | **8.71** | **8.74 (6.2-12.34)** |
| Muscle spasms | 1.17 | 1.17 (1.02–1.33) | 0.62 | 0.62 (0.54-0.71) |
| Muscular weakness | 1.40 | 1.41 (1.27–1.57) | **2.65** | **2.67 (2.31-3.09)** |
| Musculoskeletal chest pain | **2.13** | **2.13 (1.75–2.6)** | **3.65** | **3.66 (2.76-4.86)** |
| Musculoskeletal discomfort | **2.07** | **2.07 (1.69–2.53)** | **4.12** | **4.13 (3.04-5.61)** |
| Musculoskeletal pain | 0.96 | 0.96 (0.83–1.1) | **2.46** | **2.47 (2.02-3.01)** |
| Musculoskeletal stiffness | **2.32** | **2.35 (2.17–2.55)** | **4.63** | **4.72 (4.17-5.34)** |
| Myalgia | 1.64 | 1.64 (1.46–1.86) | 1.36 | 1.36 (1.19-1.56) |
| Myocardial infarction | 0.92 | 0.92 (0.84–1.01) | **2.35** | **2.37 (2.08-2.69)** |
| Nail disorder | 1.84 | 1.84 (1.45–2.34) | **2.37** | **2.37 (1.75-3.22)** |
| Nail psoriasis | **3.34** | **3.34 (2.61–4.28)** | **8.71** | **8.73 (5.55-13.73)** |
| Nasal congestion | **2.85** | **2.87 (2.55–3.22)** | **3.48** | **3.51 (3.01-4.1)** |
| Nasopharyngitis | 1.70 | 1.77 (1.7–1.84) | **3.32** | **3.54 (3.35-3.75)** |
| Nausea | 1.41 | 1.42 (1.34–1.52) | 0.19 | 0.17 (0.16-0.18) |
| Neck pain | 1.82 | 1.83 (1.61–2.08) | **3.27** | **3.29 (2.74-3.96)** |
| Needle issue | 0.63 | 0.63 (0.51–0.78) | **53.89** | **54.02 (17.15-170.14)** |
| Neoplasm malignant | 0.94 | 0.94 (0.78–1.13) | 1.54 | 1.54 (1.22-1.96) |
| Nephrolithiasis | 1.56 | 1.56 (1.36–1.79) | **2.53** | **2.54 (2.11-3.06)** |
| Nervousness | 1.93 | 1.94 (1.64–2.29) | **2.36** | **2.37 (1.92-2.94)** |
| Neuropathy peripheral | 0.85 | 0.85 (0.69–1.04) | **2.30** | **2.3 (1.71-3.09)** |
| Night sweats | 1.28 | 1.28 (1.09–1.51) | **2.32** | **2.32 (1.86-2.9)** |
| Ocular hyperaemia | **2.65** | **2.65 (2.07–3.4)** | **4.23** | **4.24 (2.95-6.08)** |
| Oedema | **2.88** | **2.89 (2.44–3.42)** | **8.78** | **8.82 (6.41-12.15)** |
| Oedema peripheral | 1.67 | 1.68 (1.52–1.85) | **3.26** | **3.3 (2.86-3.8)** |
| Oesophageal candidiasis | **5.66** | **5.67 (4.24–7.58)** | **18.86** | **18.91 (9.93-36.01)** |
| Off label use | 0.36 | 0.35 (0.32–0.39) | 1.35 | 1.35 (1.2-1.53) |
| Oral candidiasis | **5.98** | **6.04 (5.23–6.99)** | **22.98** | **23.24 (16.43-32.89)** |
| Oral herpes | **2.04** | **2.05 (1.78–2.36)** | **7.68** | **7.73 (5.88-10.14)** |
| Oral pain | **5.70** | **5.72 (4.41–7.42)** | **7.58** | **7.6 (5.17-11.18)** |
| Oropharyngeal discomfort | **4.00** | **4.01 (3.01–5.35)** | **6.49** | **6.5 (4.17-10.13)** |
| Oropharyngeal pain | **2.44** | **2.51 (2.37–2.66)** | **6.81** | **7.1 (6.41-7.86)** |
| Osteoarthritis | 0.59 | 0.59 (0.51–0.68) | **5.41** | **5.44 (4.13-7.16)** |
| Osteomyelitis | 1.23 | 1.24 (1.02–1.49) | **2.99** | **3 (2.28-3.95)** |
| Osteoporosis | 1.26 | 1.26 (1.05–1.51) | **5.05** | **5.07 (3.67-6.99)** |
| Pain | **2.04** | **2.14 (2.05–2.23)** | **3.15** | **3.37 (3.18-3.56)** |
| Pain in extremity | 1.70 | 1.73 (1.64–1.83) | **3.60** | **3.73 (3.44-4.03)** |
| Pain of skin | **3.19** | **3.21 (2.73–3.76)** | **8.29** | **8.34 (6.24-11.14)** |
| Pallor | 1.07 | 1.07 (0.85–1.34) | **2.31** | **2.32 (1.7-3.16)** |
| Palpitations | 1.37 | 1.37 (1.14–1.64) | 0.86 | 0.86 (0.71-1.04) |
| Panniculitis | **2.66** | **2.66 (2.05–3.46)** | **38.17** | **38.26 (14.09-103.9)** |
| Paraesthesia | 0.96 | 0.96 (0.88–1.05) | **2.74** | **2.76 (2.42-3.15)** |
| Pemphigus | 1.97 | 1.97 (1.68–2.31) | NA | NA |
| Pericarditis | 1.66 | 1.66 (1.32–2.1) | **32.33** | **32.41 (13.22-79.45)** |
| Peripheral swelling | 1.76 | 1.81 (1.73–1.9) | **4.10** | **4.31 (4.01-4.63)** |
| Pharyngeal oedema | 1.81 | 1.81 (1.49–2.21) | **4.24** | **4.25 (3.12-5.8)** |
| Pharyngitis | **2.22** | **2.23 (1.99–2.5)** | **9.43** | **9.52 (7.53-12.04)** |
| Pharyngitis streptococcal | 1.45 | 1.45 (1.29–1.64) | **4.85** | **4.88 (3.97-6)** |
| Pigmentation disorder | **13.74** | **13.79 (9.59–19.82)** | **11.45** | **11.49 (7.21-18.31)** |
| Pneumonia | 1.20 | 1.21 (1.15–1.27) | **3.24** | **3.37 (3.14-3.62)** |
| Poor quality sleep | 1.41 | 1.41 (1.12–1.78) | 1.56 | 1.56 (1.18-2.05) |
| Postoperative wound infection | 0.72 | 0.72 (0.58–0.89) | **8.82** | **8.83 (5.36-14.56)** |
| Product administration error | 1.95 | 1.96 (1.56–2.45) | **3.80** | **3.81 (2.73-5.33)** |
| Product availability issue | **4.78** | **4.79 (3.56–6.45)** | **26.95** | **27.01 (11.87-61.46)** |
| Product dose omission issue | 0.72 | 0.71 (0.67–0.75) | 0.39 | 0.37 (0.35-0.39) |
| Product prescribing error | **9.31** | **9.41 (7.92–11.18)** | **34.29** | **34.67 (22.62-53.15)** |
| Product storage error | 0.86 | 0.86 (0.77–0.97) | **84.33** | **84.96 (37.9-190.48)** |
| Product use in unapproved indication | **4.67** | **4.7 (4.05–5.47)** | **8.65** | **8.72 (6.79-11.2)** |
| Product use issue | 0.62 | 0.62 (0.55–0.7) | **4.69** | **4.71 (3.76-5.91)** |
| Productive cough | 1.93 | 1.94 (1.67–2.25) | **2.99** | **3.01 (2.44-3.7)** |
| Prostate cancer | 0.61 | 0.61 (0.51–0.73) | **2.08** | **2.08 (1.63-2.67)** |
| Pruritus | **2.93** | **3.08 (2.93–3.23)** | **3.10** | **3.25 (3.06-3.46)** |
| Psoriasis | 1.59 | 1.92 (1.88–1.97) | 1.18 | 1.28 (1.25-1.31) |
| Psoriatic arthropathy | 1.55 | 1.66 (1.61–1.71) | 1.22 | 1.26 (1.22-1.3) |
| Pulmonary congestion | **2.74** | **2.75 (2.25–3.35)** | **3.04** | **3.05 (2.37-3.93)** |
| Pulmonary embolism | 1.06 | 1.06 (0.91–1.24) | **2.75** | **2.76 (2.19-3.48)** |
| Pulmonary oedema | 0.62 | 0.62 (0.51–0.75) | **3.10** | **3.11 (2.26-4.27)** |
| Pustular psoriasis | 1.22 | 1.22 (1.05–1.42) | **2.78** | **2.79 (2.25-3.46)** |
| Pyelonephritis | **2.29** | **2.3 (1.8–2.92)** | **5.99** | **6 (3.97-9.08)** |
| Pyrexia | **2.29** | **2.34 (2.19–2.5)** | **5.40** | **5.55 (4.99-6.18)** |
| Rash | 1.98 | 2.02 (1.91–2.14) | 1.46 | 1.48 (1.39-1.57) |
| Rash erythematous | 1.38 | 1.38 (1.16–1.65) | **3.07** | **3.07 (2.37-3.99)** |
| Rash macular | 1.27 | 1.27 (1.08–1.49) | 1.77 | 1.77 (1.45-2.18) |
| Rash pruritic | 1.32 | 1.32 (1.13–1.54) | 1.51 | 1.51 (1.26-1.82) |
| Rash pustular | 1.57 | 1.58 (1.32–1.88) | **4.02** | **4.03 (3.06-5.31)** |
| Rebound psoriasis | **9.88** | **9.91 (7.29–13.48)** | 1.75 | 1.75 (1.4-2.2) |
| Rectal haemorrhage | 1.78 | 1.78 (1.45–2.19) | 1.87 | 1.87 (1.46-2.4) |
| Red blood cell sedimentation rate increased | **3.01** | **3.01 (2.38–3.82)** | **6.27** | **6.28 (4.27-9.24)** |
| Renal disorder | 1.21 | 1.21 (0.98–1.5) | 1.35 | 1.36 (1.05-1.75) |
| Renal failure | 0.74 | 0.74 (0.61–0.91) | 1.35 | 1.35 (1.05-1.73) |
| Renal impairment | 1.18 | 1.18 (0.96–1.47) | 1.28 | 1.29 (1-1.65) |
| Respiratory disorder | 1.49 | 1.5 (1.19–1.89) | 1.28 | 1.28 (0.98-1.66) |
| Respiratory tract infection | 1.50 | 1.51 (1.34–1.69) | **2.01** | **2.02 (1.74-2.34)** |
| Rheumatic fever | 0.96 | 0.96 (0.8–1.17) | **50.90** | **51.05 (18.89-137.99)** |
| Rheumatoid arthritis | 1.23 | 1.23 (1.13–1.34) | **6.05** | **6.14 (5.23-7.2)** |
| Rheumatoid nodule | **2.99** | **3 (2.39–3.75)** | **18.34** | **18.39 (10.21-33.14)** |
| Rhinitis | **3.41** | **3.43 (2.84–4.13)** | **9.95** | **10 (6.97-14.34)** |
| Rhinorrhoea | **2.99** | **3.02 (2.71–3.36)** | **2.76** | **2.79 (2.45-3.18)** |
| Rib fracture | 1.21 | 1.21 (1–1.47) | **3.12** | **3.13 (2.35-4.16)** |
| Road traffic accident | 0.75 | 0.75 (0.62–0.91) | **2.45** | **2.46 (1.86-3.24)** |
| SARS-CoV-2 test positive | **2.75** | **2.76 (2.17–3.5)** | **5.10** | **5.11 (3.54-7.37)** |
| Scab | **3.10** | **3.11 (2.57–3.76)** | **6.56** | **6.59 (4.8-9.04)** |
| Scar | **2.10** | **2.11 (1.81–2.45)** | **12.42** | **12.49 (8.75-17.83)** |
| Sciatica | 1.19 | 1.19 (1–1.42) | **3.56** | **3.57 (2.72-4.69)** |
| Scratch | **3.10** | **3.12 (2.68–3.63)** | **12.27** | **12.36 (8.9-17.16)** |
| Seasonal allergy | 1.55 | 1.56 (1.34–1.81) | 1.76 | 1.76 (1.47-2.11) |
| Secretion discharge | **2.40** | **2.4 (1.9–3.04)** | **4.90** | **4.91 (3.39-7.09)** |
| Sepsis | 1.20 | 1.21 (1.05–1.38) | **2.33** | **2.34 (1.94-2.82)** |
| Septic shock | 1.39 | 1.39 (1.1–1.76) | 1.74 | 1.74 (1.31-2.32) |
| Sinus congestion | 1.09 | 1.09 (0.88–1.36) | **2.86** | **2.87 (2.09-3.94)** |
| Sinus disorder | 1.44 | 1.44 (1.17–1.77) | 1.32 | 1.32 (1.04-1.68) |
| Sinusitis | 1.41 | 1.43 (1.36–1.5) | **2.68** | **2.77 (2.58-2.97)** |
| Sjogren's syndrome | 1.64 | 1.65 (1.32–2.05) | **6.42** | **6.43 (4.26-9.7)** |
| Skin burning sensation | **2.74** | **2.75 (2.38–3.17)** | **3.25** | **3.27 (2.71-3.94)** |
| Skin cancer | 0.64 | 0.64 (0.53–0.76) | 1.48 | 1.48 (1.17-1.87) |
| Skin discolouration | 1.93 | 1.94 (1.71–2.2) | **3.95** | **3.98 (3.29-4.81)** |
| Skin disorder | 1.75 | 1.76 (1.54–2) | **3.09** | **3.1 (2.59-3.72)** |
| Skin exfoliation | **3.88** | **3.96 (3.63–4.32)** | **4.73** | **4.83 (4.28-5.44)** |
| Skin fissures | **4.34** | **4.37 (3.79–5.04)** | **5.35** | **5.4 (4.42-6.6)** |
| Skin haemorrhage | **3.42** | **3.46 (3.12–3.83)** | **5.18** | **5.25 (4.51-6.12)** |
| Skin infection | 1.31 | 1.31 (1.12–1.53) | **3.83** | **3.84 (2.99-4.93)** |
| Skin injury | **3.06** | **3.06 (2.34–4)** | **19.46** | **19.5 (9.5-40.04)** |
| Skin irritation | 1.65 | 1.65 (1.37–1.98) | **2.61** | **2.62 (2.04-3.36)** |
| Skin laceration | 0.48 | 0.48 (0.39–0.58) | 1.81 | 1.81 (1.38-2.37) |
| Skin lesion | **5.07** | **5.13 (4.52–5.81)** | **8.23** | **8.33 (6.83-10.17)** |
| Skin mass | **2.45** | **2.45 (1.9–3.17)** | **12.85** | **12.88 (7.08-23.41)** |
| Skin plaque | **2.94** | **2.97 (2.67–3.29)** | **19.81** | **20.1 (15.12-26.73)** |
| Skin ulcer | 1.52 | 1.53 (1.34–1.74) | **2.36** | **2.37 (1.99-2.81)** |
| Sleep apnoea syndrome | 0.94 | 0.94 (0.8–1.11) | **4.29** | **4.3 (3.25-5.69)** |
| Sleep disorder | **2.24** | **2.24 (1.86–2.7)** | **2.21** | **2.22 (1.77-2.77)** |
| Sneezing | 1.90 | 1.91 (1.53–2.37) | **2.57** | **2.58 (1.94-3.43)** |
| Somnolence | 1.67 | 1.68 (1.5–1.88) | 1.70 | 1.7 (1.49-1.95) |
| Speech disorder | **2.12** | **2.13 (1.88–2.42)** | **6.36** | **6.41 (5.13-8.01)** |
| Spinal pain | **3.27** | **3.3 (2.94–3.7)** | **9.96** | **10.08 (8.04-12.63)** |
| Staphylococcal infection | 1.21 | 1.22 (1.05–1.4) | **4.37** | **4.39 (3.45-5.59)** |
| Stomatitis | **2.99** | **3 (2.47–3.64)** | **2.91** | **2.91 (2.29-3.7)** |
| Stress | 1.90 | 1.92 (1.75–2.11) | 1.68 | 1.7 (1.52-1.89) |
| Suspected COVID-19 | **2.22** | **2.22 (1.76–2.8)** | **9.13** | **9.15 (5.7-14.69)** |
| Swelling | 1.66 | 1.67 (1.52–1.84) | **4.63** | **4.69 (4.01-5.48)** |
| Swelling face | 1.79 | 1.8 (1.63–1.99) | **3.44** | **3.47 (3-4.02)** |
| Swollen tongue | **2.29** | **2.3 (1.96–2.71)** | **4.01** | **4.02 (3.17-5.11)** |
| Syncope | 0.99 | 0.99 (0.88–1.1) | **2.01** | **2.02 (1.73-2.34)** |
| Synovitis | **2.79** | **2.8 (2.38–3.29)** | **29.62** | **29.8 (17.42-50.98)** |
| Systemic lupus erythematosus | 0.83 | 0.83 (0.75–0.91) | **5.82** | **5.88 (4.86-7.11)** |
| Tenderness | **4.53** | **4.54 (3.43–5.99)** | **4.69** | **4.7 (3.26-6.77)** |
| Tendonitis | 1.87 | 1.88 (1.62–2.18) | **4.52** | **4.55 (3.6-5.74)** |
| Therapeutic product effect decreased | 0.82 | 0.82 (0.75–0.89) | 1.55 | 1.55 (1.39-1.74) |
| Therapeutic product effect delayed | **2.43** | **2.43 (1.92–3.08)** | **8.23** | **8.25 (5.24-13)** |
| Therapeutic product effect incomplete | 1.75 | 1.78 (1.67–1.89) | **4.44** | **4.56 (4.14-5.04)** |
| Therapeutic response decreased | 0.83 | 0.83 (0.71–0.96) | 0.67 | 0.67 (0.57-0.78) |
| Therapeutic response shortened | **4.49** | **4.53 (3.91–5.24)** | **53.89** | **54.41 (30.65-96.56)** |
| Therapy cessation | 0.81 | 0.8 (0.7–0.93) | 1.44 | 1.44 (1.2-1.72) |
| Therapy interrupted | 0.78 | 0.78 (0.67–0.9) | **3.30** | **3.31 (2.62-4.18)** |
| Therapy non-responder | 0.80 | 0.8 (0.69–0.93) | 0.27 | 0.27 (0.23-0.31) |
| Throat irritation | 1.62 | 1.62 (1.42–1.85) | **4.13** | **4.15 (3.36-5.14)** |
| Throat tightness | 1.87 | 1.87 (1.52–2.31) | **5.52** | **5.53 (3.86-7.92)** |
| Thrombosis | 0.89 | 0.89 (0.72–1.08) | **2.46** | **2.46 (1.84-3.3)** |
| Tinea pedis | **4.41** | **4.42 (3.35–5.82)** | **17.96** | **18.01 (9.45-34.33)** |
| Tinnitus | 0.82 | 0.82 (0.66–1.02) | 0.73 | 0.73 (0.57-0.92) |
| Tonsillitis | **2.21** | **2.22 (1.88–2.62)** | **17.81** | **17.9 (11.34-28.26)** |
| Tooth abscess | 1.41 | 1.42 (1.22–1.65) | **4.94** | **4.96 (3.82-6.43)** |
| Tooth infection | 0.91 | 0.91 (0.78–1.07) | **2.50** | **2.51 (2-3.14)** |
| Transient ischaemic attack | 0.84 | 0.84 (0.69–1.03) | **2.36** | **2.37 (1.78-3.16)** |
| Treatment failure | **2.33** | **2.34 (2.07–2.65)** | **4.02** | **4.05 (3.38-4.85)** |
| Tremor | 0.94 | 0.94 (0.78–1.14) | 0.48 | 0.47 (0.39-0.57) |
| Tuberculosis | 1.12 | 1.12 (0.92–1.36) | **3.96** | **3.97 (2.88-5.48)** |
| Type 2 diabetes mellitus | 1.07 | 1.07 (0.92–1.24) | **6.15** | **6.17 (4.61-8.27)** |
| Ulcer | 1.97 | 1.98 (1.55–2.51) | **2.65** | **2.65 (1.94-3.63)** |
| Underdose | 0.34 | 0.34 (0.28–0.41) | **22.45** | **22.51 (11-46.05)** |
| Upper limb fracture | 1.24 | 1.25 (1.04–1.49) | **2.45** | **2.46 (1.92-3.16)** |
| Upper respiratory tract infection | 1.57 | 1.58 (1.47–1.7) | 1.16 | 1.16 (1.07-1.26) |
| Urinary tract infection | 1.39 | 1.4 (1.32–1.48) | **3.29** | **3.38 (3.1-3.69)** |
| Urticaria | 1.59 | 1.61 (1.5–1.73) | **3.00** | **3.06 (2.77-3.38)** |
| Uveitis | 1.92 | 1.92 (1.6–2.3) | **13.92** | **13.98 (8.81-22.17)** |
| Vertigo | 0.99 | 0.99 (0.85–1.15) | 1.25 | 1.25 (1.05-1.5) |
| Viral infection | 1.41 | 1.42 (1.21–1.66) | **3.95** | **3.97 (3.09-5.09)** |
| Vision blurred | 0.90 | 0.9 (0.8–1.02) | **2.24** | **2.25 (1.89-2.67)** |
| Visual impairment | 0.87 | 0.87 (0.74–1.02) | **3.07** | **3.08 (2.41-3.93)** |
| Vomiting | 1.51 | 1.52 (1.39–1.66) | 0.43 | 0.42 (0.38-0.45) |
| Weight decreased | 1.55 | 1.55 (1.43–1.7) | 0.46 | 0.45 (0.42-0.49) |
| Weight increased | 1.19 | 1.19 (1.07–1.32) | **2.91** | **2.94 (2.51-3.43)** |
| Wheezing | **3.64** | **3.65 (2.93–4.55)** | **5.50** | **5.52 (3.98-7.64)** |
| Wound | **2.75** | **2.79 (2.54–3.05)** | **13.53** | **13.78 (11.16-17.02)** |
| Wound infection | 1.26 | 1.26 (1.06–1.51) | **7.57** | **7.6 (5.26-10.98)** |
| Wrong technique in device usage process | **6.14** | **6.15 (4.6–8.24)** | NA | NA |
| Wrong technique in product usage process | 0.20 | 0.2 (0.18–0.22) | **5.27** | **5.29 (4.16-6.74)** |

ROR, reporting odds ratio; PRR, proportional reporting ratio; CI, confidence interval.

*Only preferred terms reported in ≥100 individual case safety reports have been included in the table.
